# PFASGroups: An Open-Source Framework for Automated Identification, Structural Classification, and Prioritization of Per- and Polyfluoroalkyl Substances

**DOI:** 10.1021/acs.jcim.6c01058

**Published:** 2026-07-02

**Authors:** Luc T. Miaz, Ian T. Cousins, Ida Rahu

**Affiliations:** Department of Environmental Science, 7675 Stockholm University, Svante Arrhenius Väg 8, SE-106 91 Stockholm, Sweden

## Abstract

Per- and polyfluoroalkyl substances (PFAS) are highly persistent synthetic chemicals that are widely detected in environmental and biological systems and are increasingly targeted by global regulatory actions. Their structural diversity, together with multiple and evolving PFAS definitions, complicates automated identification and classification across large chemical data sets. Here we present *PFASGroups*, an open-source cheminformatics framework for automated identification, structural classification, and prioritization of PFAS, designed for seamless integration into machine learning (ML) workflows. *PFASGroups* combines SMARTS-based functional group detection with graph-based analysis of per- and polyfluorinated components to assign PFAS groups and characterize their size, topology, and structural context. The framework implements an extensible library of PFAS group definitions, including OECD-derived classes, common organic functional groups, and fluorotelomer-specific patterns, and supports evaluation of multiple regulatory PFAS definitions (OECD, EU, OPPT 2023, UK, PFASSTRUCTv5). Across benchmark data sets, *PFASGroups* demonstrates computational performance suitable for large-scale screening and shows high agreement with existing tools, with systematic differences reflecting broader structural coverage and multigroup assignment. The graph-based representation enables robust handling of complex and highly branched molecules while maintaining high specificity in group detection. *PFASGroups*’ identification and descriptor generation capabilities are readily usable in ML pipelines and for automatic prioritization. The module produces structural embeddings that integrate functional and graph descriptors, thereby improving predictive modeling of toxicological end points compared to conventional PFAS-specific fingerprints. Application to large chemical inventories, including the ECHA Classification and Labeling database, demonstrates its utility for high-throughput PFAS screening and regulatory decision support. *PFASGroups* is available as open-source software via GitHub (https://github.com/lucmiaz/PFASGroups) and PyPI, with a complementary browser-based interface for rapid PFAS screening.

## Graphical abstract

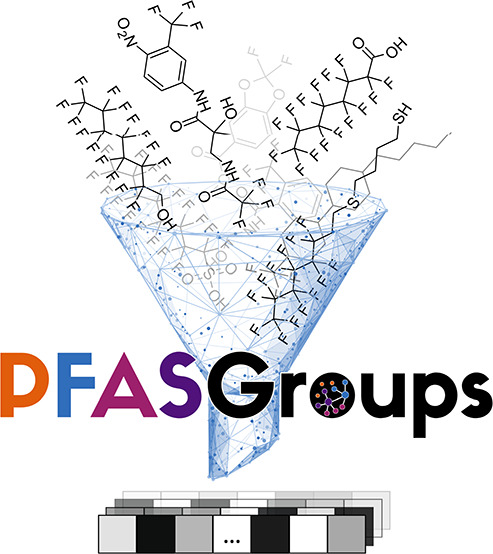

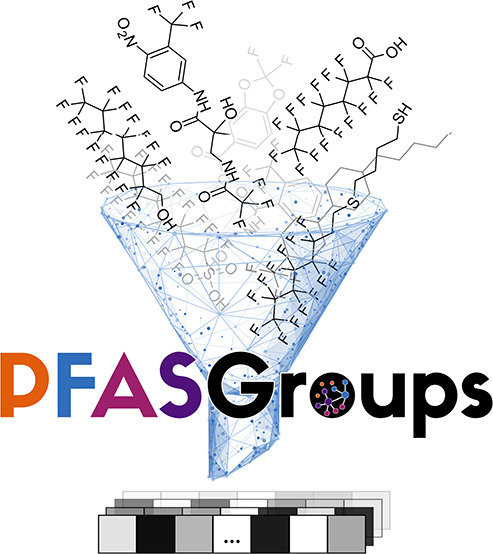

## Introduction

Per- and polyfluoroalkyl substances (PFAS) are a broad class of synthetic organofluorine compounds defined by the Organisation for Economic Co-operation and Development (OECD) as substances containing, with few noted exceptions, at least one fully fluorinated methyl (−CF_3_) or methylene (−CF_2_−) carbon atom.^1^ Despite this small shared structural moiety, PFAS encompass highly diverse molecular structures ranging from small molecules to large fluorinated polymers.^2^ Carbon–fluorine bonds impart exceptional chemical and thermal stability and distinctive interfacial behavior, properties that have enabled widespread industrial and commercial use.^3^ These same features, however, contribute to environmental persistence and resistance to degradation, resulting in global distribution and accumulation across environmental compartments and biological systems. Knowledge of the extreme environmental persistence of all PFAS, together with growing evidence of mobility, bioaccumulation potential, and adverse health effects for specific PFAS subclasses^4–7^ has intensified regulatory attention, including proposals in several jurisdictions to regulate PFAS collectively as a chemical class.^8–10^

Understanding the structural composition and extent of the PFAS universe has therefore become increasingly important. Although curated inventories such as the OECD PFAS list (4729 substances)^11^ and the Swedish Chemicals Agency’s PRIO database (∼14000 substances)^12^ provide important resources for identification and regulatory assessment, they likely capture only a fraction of the potential PFAS chemical space. Recent analyses suggest that chemical databases such as PubChem may contain more than seven million structures that could satisfy PFAS definitions, depending on the criteria applied.^13^ Concurrently, advances in high-resolution mass spectrometry and nontarget screening continue to reveal previously unrecognized PFAS in environmental samples.^14–18^

To support consistent identification and communication across this expanding chemical space, several efforts have aimed to organize PFAS into structural groups and subclasses. The OECD report^1^ provides a widely adopted framework that categorizes PFAS according to characteristic structural features and functional groups. However, applying these definitions manually across large chemical data sets is impractical, motivating the development of computational approaches for PFAS categorization.

One early effort to apply cheminformatics to systematic PFAS categorization was the splitPFAS^19^ workflow, which subdivides PFAS structures following a C_*n*_F_2*n*+1_–X–R pattern to enable structure-based grouping of PFAS molecules. The PubChem PFAS Tree^13^ initiative applied the OECD PFAS definition to the PubChem database and introduced a classification browser for navigating large collections of PFAS structures. PFAS-specific structural fingerprints have also been developed, including the TxP_PFAS^20^ chemotypes derived from the ToxPrint framework, which encode characteristic fluorinated chains and bonding patterns for computational profiling of PFAS inventories. Another approach, PFAS-Atlas^21^ combines rule-based classification with machine learning (ML) models to automatically group PFAS structures and visualize PFAS chemical space.

These approaches support applications such as structural grouping, read-across, prioritization for toxicological evaluation, and ML-based property prediction.^22–24^ However, existing approaches are often limited to predefined fingerprints, fixed structural taxonomies, or database-specific classification workflows. Consequently, they typically provide limited information on the size, topology, and structural context of fluorinated components within molecules. In addition, many currently available tools are not readily designed for flexible application across arbitrary chemical data sets, integration of multiple regulatory PFAS definitions, or the generation of graph-aware molecular representations suitable for downstream ML applications.

Here we present *PFASGroups*, an open-source cheminformatics framework for automated PFAS identification, structural classification, and embedding generation across diverse chemical data sets. *PFASGroups* combines SMARTS-based functional group detection with graph-based analysis of fluorinated components to jointly characterize PFAS structural groups and fluorinated substructure topology. In contrast to predefined PFAS fingerprints or fixed classification schemes, the framework generates flexible graph-aware embeddings that capture both the presence and structural organization of fluorinated components in molecular graphs. *PFASGroups* further integrates multiple regulatory PFAS definitions within a unified workflow and provides both a Python package and browser-based web application for scalable screening and prioritization of large chemical inventories.

## Implementation

### Algorithm Overview

*PFASGroups* identifies fluorinated structural components and associated functional groups defining PFAS subclasses (hereafter PFAS groups) from structures provided as SMILES, InChI, or *RDKit* ^25^ molecule objects. Because detection is independent of regulatory PFAS definitions, identified structures may fall outside specific definitions such as OECD criteria; definition-based classification is therefore handled separately (see Step 6).

*PFASGroups* is implemented within *HalogenGroups*, a general framework for detecting per- and polyhalogenated structural moieties across multiple halogens. As a fluorine-specific configuration, it provides PFAS group definitions and structural metrics tailored to fluorinated compounds. Through the *HalogenGroups* wrapper package, the same component-detection and classification workflow can also be applied to chlorine-, bromine-, and iodine-containing molecules (see Supporting Information (SI), Section S1).

The analysis proceeds through six sequential steps: preprocessing of input structures, identification of fluorinated components, PFAS group evaluation and detection, component association and validation, and result assembly (Figure 1).

**Figure 1:**
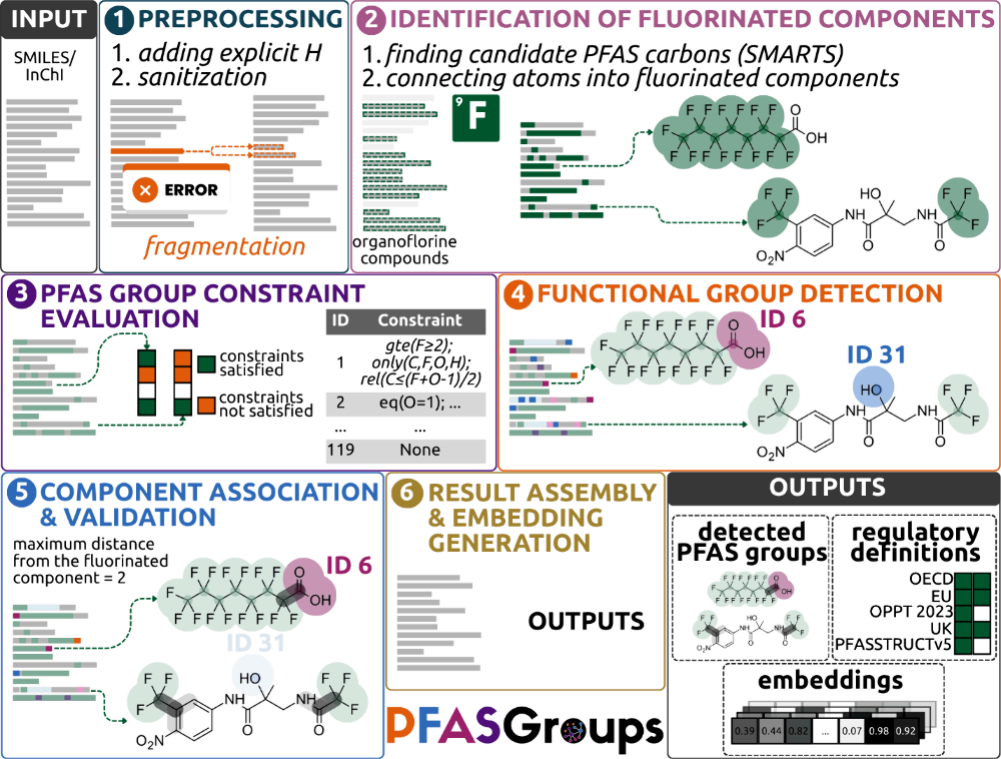
Workflow of the *PFASGroups* algorithm for identifying fluorinated structural components and associated PFAS groups. Input molecular structures are first preprocessed and analyzed to identify fluorinated components using SMARTS patterns and graph connectivity. Candidate PFAS groups are then evaluated using predefined molecular constraints, followed by SMARTS-based functional group detection and validation of their association with fluorinated components based on graph-distance criteria. Detected PFAS groups and associated components are subsequently assembled into structural embeddings that combine functional-group information with graph-derived descriptors of fluorinated components. The workflow additionally enables the evaluation of several regulatory PFAS definitions implemented in *PFASGroups*.

#### Step 1: Molecule Preprocessing

Input structures are converted to *RDKit* molecule objects with explicit hydrogens and sanitized using *RDKit*’s standard sanitization procedure to ensure chemically consistent molecular graphs. Structures containing multiple disconnected fragments (e.g., salts) are automatically separated into individual fragments, each of which is processed independently through the *PFASGroups* workflow.

If sanitization fails, the structure is also decomposed into fragments, and the valid fragments are analyzed independently. Sanitization failures are rare (17 out of 208266 during analysis of ECHA’s Classification and Labeling (C&L) inventory) and typically arise from unusual valence states or uncommon bonding patterns unrelated to fluorinated PFAS moieties. Because *PFASGroups* targets well-defined fluorinated substructures, analyzing valid fragments does not affect the identification of PFAS components, and the deterministic fragmentation procedure ensures reproducible results during batch screening.

#### Step 2: Identification of Fluorinated Components

After preprocessing, structures lacking a fluorine atom are excluded from further analysis, reducing unnecessary computation when screening large chemical libraries.

For the remaining structures, fluorinated components are identified by analyzing the molecular graph. The *RDKit* representation is converted into a *NetworkX* ^26^ graph, with atoms as nodes and bonds as edges. Candidate atoms belonging to fluorinated substructures are detected using predefined *componentSMARTS* patterns describing characteristic per- and polyfluorinated moieties (SI Section S2). Adjacent candidate atoms are merged using graph connectivity analysis to form contiguous fluorinated components representing individual fluorinated regions within the structure. These components subsequently serve as structural anchors for PFAS group detection. By operating directly on molecular connectivity, the graph-based approach enables consistent characterization of linear, branched, and cyclic fluorinated structures without relying on specialized rules for linearized representations such as SMILES.^27^

#### Step 3: PFAS Group Constraint Evaluation

After fluorinated components have been identified, *PFASGroups* evaluates predefined PFAS groups corresponding to functional groups associated with those components. The default *PFASGroups* library contains 119 PFAS groups, organized into three categories:

- *OECD PFAS groups* (28 groups; IDs 1–28), corresponding to compound classes defined in the OECD PFAS terminology report.^1^
- *Common organic functional groups* (48 groups; IDs 29–76), representing widely occurring functional groups such as alcohols, ketones, amides, and ethers.
- *Fluorotelomer PFAS groups* (43 groups; IDs 77–119), describing compounds in which a functional group is connected to a fluorinated chain through a characteristic −CH_2_– linker.

Complete specifications for all PFAS group definitions, including SMARTS patterns and associated constraints, are provided in Section S3 of the Supporting Information. Furthermore, additional groups can be incorporated using the same framework.

Before SMARTS-based matching, *PFASGroups* evaluates optional molecular formula constraints associated with each PFAS group. These constraints act as a computationally efficient prefilter, excluding chemically incompatible structures before more expensive pattern-matching operations.

Supported constraint types include relative elemental ratios (*rel*), minimum or maximum element counts (*gte*/*lte*), exact element counts (*eq*), and element-restriction lists (*only*). For example, the definition of OECD perfluoroalkyl carboxylic acids (PFCAs, ID 6) requires exactly two oxygen atoms and restricts the structure to the elements C, F, O, and H. Structures failing the defined constraints are excluded from further evaluation for the corresponding PFAS group.

#### Step 4: Functional Group Detection

For PFAS groups that pass the constraint evaluation, functional groups are detected using the SMARTS patterns associated with each PFAS group definition, employing *RDKit*’s substructure search functionality.

Each successful match returns the atom indices corresponding to a detected functional group instance. *PFASGroups* then verifies whether the number of detected matches satisfies the requirements defined for the corresponding PFAS group. While many PFAS groups are defined by a single functional group occurrence, some require multiple instances of the same functional group. For example, perfluoroalkyl dicarboxylic acids (PFdiCAs, ID 8) require two carboxylic acid groups associated with the fluorinated component. Only PFAS groups whose pattern requirements are satisfied are retained for the subsequent validation step.

#### Step 5: Component Association and Validation

Detected functional groups are associated with previously identified fluorinated components by evaluating whether they occur within a user-defined bond-distance threshold (*max_dist_from_comp*) from the component (see SI, Section S3.2).

This threshold defines the maximum bond distance at which a functional group is still considered associated with a fluorinated component. Such flexibility is important because functional groups in PFAS molecules are often linked to fluorinated chains via short linkers or spacer units, which influence physicochemical properties and environmental behavior.

Association is evaluated by examining the graph distances between atoms of the functional group and those of the fluorinated component. Starting from direct adjacency, the search iteratively expands to increasing bond distances up to the specified threshold. Functional groups located within this distance are considered associated with the corresponding fluorinated component.

This validation is also required for PFAS groups defined by multiple functional groups. For example, PFdiCAs (ID 8) require two carboxylic acid groups associated with the same fluorinated component. If the detected functional groups are separated from the fluorinated chain by more than the allowed distance or belong to different components, the PFAS group definition is not satisfied.

Additional validation rules are applied for specific PFAS subclasses. For example, fluorotelomer PFAS groups require verification of a characteristic – CH_2_– linker connecting the functional group to the fluorinated chain, which is evaluated using predefined *linker_smarts* patterns (SI, Section S3.2).

#### Step 6: Result Assembly and Embedding Generation

In the final step, detected PFAS groups and their associated fluorinated components are assembled into a structured representation. *PFASGroups* returns a *PFASEmbeddingSet*, a list-like container holding one *PFASEmbedding* object for each analyzed structure (SI, Section S4.3). Each embedding stores the detected PFAS groups, their associated fluorinated components, and the atom indices corresponding to the SMARTS matches defining each group.

*PFASEmbedding* objects can be converted to *NumPy* feature vectors using the *to_array­()* method, enabling direct use in downstream analyses such as clustering, dimensionality reduction, similarity searches, or ML workflows.

By default, the embedding contains one feature per PFAS group, excluding the aggregated telomer group and the selected halogen group to avoid redundant features (117 groups when *halogens = ‘F’*). Alternatively, it can be restricted via the *group_selection* parameter to specific PFAS groups described in Step 3. When multiple halogens are analyzed simultaneously (e.g., *halogens = [‘F’,‘Cl’]*), separate vectors are generated for each halogen and concatenated into a combined feature matrix, with feature names suffixed by the corresponding halogen.

Unlike conventional chemical fingerprints, which encode only the presence or absence of structural moieties, *PFASGroups* embeddings can capture quantitative and structural information about fluorinated components associated with each PFAS group. For example, the PFAS group corresponding to alcohol functional groups (ID 31) would produce a binary value of 1 in a traditional fingerprint if at least one – OH group is present. In *PFASGroups*, the embedding can additionally encode the number of fluorinated components associated with that functional group. Thus, a structure containing two distinct fluorinated components, each bearing an alcohol group, would yield a value of 2 for this descriptor.

In addition to such count-based and binary descriptors, *PFASGroups* embeddings can also incorporate graph-theoretical descriptors characterizing the topology of fluorinated components. These include component size, the number of – CF_2_– units, branching indices describing chain linearity, graph diameter and radius, effective graph resistance, and the fraction of carbon atoms in the structure belonging to the fluorinated component. Node sets corresponding to key structural positions within each component (center, periphery, and barycenter) are further used to calculate distance metrics relating functional groups to the fluorinated backbone.

At the molecule level, additional metrics summarize structural properties across all detected fluorinated components, including component counts, largest-component size, cumulative number of fluorinated carbon atoms, and aggregate topology metrics. The specific descriptors included in the embedding can be configured through the *component_metrics* and *molecule_metrics* options. Mathematical definitions and computational details of these descriptors are provided in the SI Section S4.

*PFASGroups* additionally evaluates several regulatory PFAS definitions using independent SMARTS-based rules, enabling streamlined comparison of classification results across regulatory frameworks. The current implementation includes five definitions: the OECD PFAS definition,^28^ the EU PFAS restriction proposal,^29^ the US EPA OPPT (2023) definition,^30,31^ the UK PFAS definition,^32^ and the PFASSTRUCTv5 definition.^33^ These definitions differ in their structural criteria, ranging from simple CF_3_/CF_2_ requirements to more complex fluorinated-carbon and heteroatom-linkage rules. Detailed SMARTS patterns and implementation rules for these definitions are provided in the SI, Section S5.

### Prioritization of PFAS Structures

Building on the embedding framework, *PFASGroups* provides a prioritization module that enables ranking of PFAS structures based on their structural characteristics. Molecules can be prioritized either by similarity to a user-defined reference set, using embedding-based similarity scores, or by intrinsic properties of their fluorinated components, such as their relative size and extent within the molecular structure. This functionality supports tasks such as identifying structurally relevant analogs or highlighting compounds with prominent fluorinated features for further investigation. Implementation details and scoring procedures are provided in the SI, Section S6.

### Software Implementation

*PFASGroups* is implemented in Python (with continuous integration for v3.9 to v3.14) using RDKit for cheminformatics operations and NetworkX for graph analysis. Additional details are provided in Section S7 of the Supporting Information.

### Software Availability

*PFASGroups* provides a Python API for PFAS classification and embedding generation and is freely available as open-source software via GitHub (https://github.com/lucmiaz/PFASGroups) and PyPI. A browser-based web application is also available at https://chem.cogitopia.dev/pfasgroups-py.

## Results and Validation

*PFASGroups* was evaluated across complementary benchmarks to assess its computational performance, classification behavior, structural detection capabilities, and utility for downstream applications, including predictive modeling and prioritization tasks.

### Computational Performance and Scaling

To characterize computational performance, *PFASGroups* was benchmarked on a stress-test set of 2500 molecules spanning a wide range of sizes (9–621 non-H atoms) and fluorinated chain lengths (5–200 carbon atoms). Across all timing profiles, execution time increased approximately quadratically with molecular size (SI, Section S8.1), reflecting the cost of graph-based operations such as component identification, distance calculations, and evaluation of topological descriptors.

The contribution of graph-derived descriptors to runtime was additionally assessed by selectively disabling metric groups. Removing effective graph resistance had little impact, whereas disabling all component-level metrics reduced runtime by approximately 20%, indicating a moderate computational overhead associated with the additional structural information they provide.

Runtime was further evaluated on the fluorinated subset of ECHA’s C&L inventory (*n* = 28328). *PFASGroups* achieved an average processing time of 23.2 ms per molecule (median 17.5 ms; 95th percentile 54.4 ms) on an Intel i7–7700HQ 2.80 GHz workstation (see *System 2b* on SI, Table S5), demonstrating suitability for high-throughput screening of large chemical inventories.

Performance was additionally assessed alongside PFAS-Atlas,^21,34^ a recent automated PFAS screening platform that combines rule-based classification with ML-based grouping and visualization, and has been applied in large-scale PFAS classification workflows. *PFASGroups* was slightly faster on the smaller OECD data set (*n* = 3707), but slower on the C&L inventory and on the large-molecule stress benchmark (*n* = 1242; molecules containing ≥35 heavy atoms) (SI, Figure S6). This trend is consistent with the additional cost of explicit graph-based structural characterization in *PFASGroups*, particularly for larger and more complex structures.

Overall, *PFASGroups* provides a practical balance between computational efficiency and structural resolution for large-scale PFAS screening. The modest computational overhead introduced by graph-based descriptors is offset by the increased robustness of component detection, particularly for branched or structurally atypical PFAS that challenge existing tools.

### Classification Behavior and Validation Across Data Sets

Given the diversity of PFAS definitions and grouping frameworks, comparison across methods helps assess how these differences translate into practical identification and grouping outcomes. As noted above, *PFASGroups* is not restricted to specific PFAS definitions, and may therefore identify fluorinated structures that fall outside individual definitions; results should be interpreted in this context.

Classification behavior was compared with PFAS-Atlas on both the OECD 2018 PFAS data set and the fluorinated subset of the ECHA’s C&L inventory. On the OECD data set, there is strong agreement between *PFASGroups* generic and fluorotelomer group assignments and PFAS-Atlas Class 1 classifications, as illustrated by the Sankey diagram in Figure S7 (SI). Remaining differences primarily arise from edge cases and differences in representation, as *PFASGroups* assigns multiple groups per molecule, whereas PFAS-Atlas reports a single primary class. Multigroup assignment captures the underlying structural complexity of many PFAS and provides a richer basis for downstream analyses such as read-across, clustering, and ML-based property prediction.

On the C&L inventory, overall agreement reached 77.7% (SI, Table S7), with the majority of compounds classified consistently (20735 PFAS and 1264 non-PFAS identified by both methods). The 6216 molecules detected only by *PFASGroups* are all fluorinated compounds, including 3856 that contain fluorine as the sole halogen and 2360 that contain additional chlorine or bromine. These cases arise from the broader structural scope of *PFASGroups*, which captures minimally fluorinated aromatics, cyclic structures, and short alkyl chains through polyhalogenated group patterns that do not satisfy the stricter polyfluoroalkyl criteria applied in PFAS-Atlas. Conversely, the 113 molecules detected only by PFAS-Atlas correspond predominantly to vinylic and gem-difluoroalkene structures (fluorine bound to sp^2^ carbon), which fall outside the fluorinated sp^3^-chain scope implemented in *PFASGroups* (SI, Figure S8).

To assess robustness with respect to molecular topology, *PFASGroups* was evaluated on a synthetic data set of 300 highly branched fluorinated compounds with controlled functional group placement (SI, Section S8.2). *PFASGroups* consistently identified the expected functional group, whereas PFAS-Atlas failed to assign a specific class in 11 cases due to increased branching complexity, instead returning nonspecific classifications such as “Other PFASs” or “Unknown” (SI, Figure S9). This highlights the advantage of graph-based structural representation for handling highly branched molecules.

Group detection specificity was evaluated on the OECD data set by analyzing codetection patterns among PFAS groups. Using a predefined hierarchy of expected overlaps among groups, *PFASGroups* achieved 100% specificity, with no unexpected group co-occurrences observed. This confirms the consistency of the SMARTS definitions and validation rules implemented in the group library (SI, Section S8.2).

Detection of fluorotelomer-specific groups was further validated on 785 fluorotelomer compounds retrieved from PubChem (SI, Section S8.3). *PFASGroups* correctly identified 734 fluorotelomers (93.5%; SI, Figure S10). The 51 undetected compounds primarily comprise short-chain or structurally atypical molecules that do not meet the CH_2_ linker constraint.

Finally, the implementation of regulatory PFAS definitions (OECD, EU PFAS Restriction, OPPT 2023, UK, and PFASSTRUCTv5) was validated using curated positive and negative test sets embedded in the configuration files. All definitions performed as expected on their respective validation sets, confirming the correctness of their implementation (SI, Section S8.4).

### Predictive Performance of *PFASGroups* Embeddings

To evaluate the utility of *PFASGroups* embeddings for downstream applications, we benchmarked their performance across 15 toxicological end points from the EPA ToxCast invitroDB v4.3 data set^35–38^ (SI, Section S8.5). A subset of ∼800 organofluorine compounds was used to enable direct comparison with the 129-bit ToxPrint PFAS fingerprint (TxP_PFAS),^39,40^ which incorporates fluorine-specific ToxPrint patterns describing cycles, branching, telomers, and halogens. Classification models were evaluated using 5 × 3 repeated stratified cross-validation.

Across the 15 end points, *PFASGroups* embeddings consistently outperformed TxP_PFAS when combined with ML models (Figure 2; SI Table S8 and Figures S11–S13). The best-performing configuration, combining total fluorinated component size with molecule-level graph descriptors, achieved a mean ROC-AUC of 0.699, compared to 0.618 for TxP_PFAS, with corresponding improvements in average precision, balanced accuracy, and Matthews correlation coefficient.

**Figure 2:**
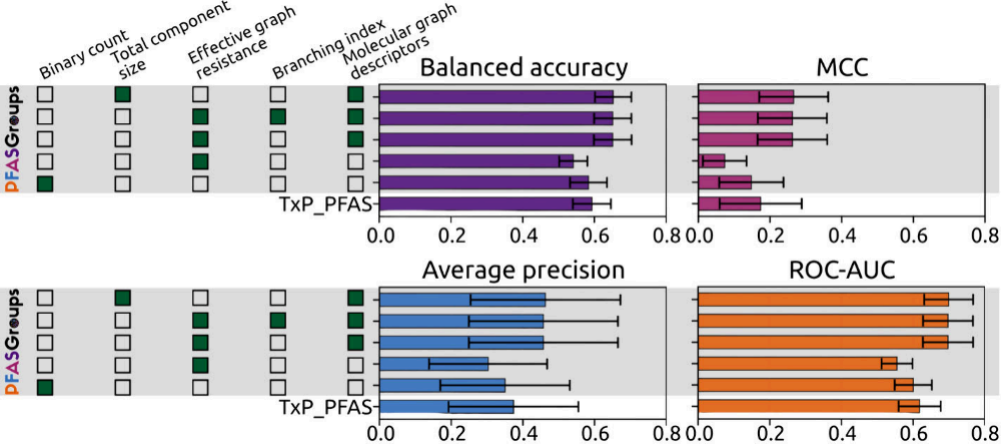
Predictive performance of PFASGroups embedding configurations across 15 ToxCast end points. Mean balanced accuracy, Matthews correlation coefficient (MCC), average precision, and ROC-AUC obtained using Gradient Boosting models and 5 × 3 repeated stratified cross-validation. Green squares indicate descriptor classes included in each embedding configuration. *PFASGroups* embeddings are compared with the PFAS-specific TxP_PFAS fingerprint. Error bars represent ±1 standard deviation across end points.

As shown in Figure 2, predictive performance was primarily driven by the inclusion of molecule-level graph descriptors, whereas alternative encodings of the same group information (e.g., binary vs effective graph resistance) had minimal impact. This suggests that capturing the size, topology, and distribution of fluorinated components is more informative than functional-group presence alone.

At the individual end point level, *PFASGroups* embeddings incorporating molecule-level metrics outperformed TxP_PFAS for the majority of end points (SI, Figure S11), with particularly large gains observed for assays such as Caspase3 HEPG2. For a small number of end points, the differences were negligible or yielded slightly higher performance for TxP_PFAS, suggesting that simpler structural fingerprints can still capture relevant patterns in some cases.

To assess the statistical significance of these differences, Bayesian correlated *t*-tests and hierarchical models^41,42^ were applied to account for dependencies introduced by repeated cross-validation. For 8 of the 15 end points, *PFASGroups* embeddings showed near-certain improvement over TxP_PFAS in ROC-AUC (posterior probability ≥ 0.95), while the remaining end points were either inconclusive or showed only weak evidence favoring TxP_PFAS (SI Figures S12 and S13).

Overall, these findings demonstrate that *PFASGroups* embeddings provide a more informative representation for predictive modeling of PFAS-related properties, particularly when incorporating graph-based descriptors that capture the structural context of fluorinated components.

## Conclusions

*PFASGroups* provides a flexible and scalable framework for PFAS identification and structural characterization across diverse chemical data sets. By combining SMARTS-based functional group detection with graph-based analysis of fluorinated components, the approach captures both the presence and structural context of PFAS-relevant features. Benchmarking demonstrated strong agreement with existing classification approaches while identifying 6216 additional PFAS candidates not detected by PFAS-Atlas and achieving a fluorotelomer detection rate of 93.5%.

As with other rule-based classification approaches, *PFASGroups* depends on the coverage of the underlying PFAS group definitions and may not explicitly classify motifs lacking a corresponding group definition. However, the framework is readily extensible through additional SMARTS-based group definitions and associated constraints. The ability to evaluate multiple regulatory definitions alongside definition-independent structural grouping further supports transparent comparison across classification frameworks.

Beyond classification, *PFASGroups* generates graph-aware embeddings that support downstream applications such as predictive modeling and structure-based prioritization. Across 15 ToxCast end points, the best-performing embedding yielded a mean ROC-AUC of 0.699 compared to 0.618 for the PFAS-specific TxP_PFAS fingerprint, with near-certain improvement observed for 8 of the 15 end points. These results highlight the value of incorporating descriptors that capture the size, topology, and distribution of fluorinated components within molecular structures.

Together, these capabilities make *PFASGroups* a versatile platform for PFAS screening, classification, prioritization, and comparative regulatory assessment across rapidly expanding chemical spaces.

## Supplementary Material



## Data Availability

The code and software for this study (v.3.2.2) are openly accessible on https://github.com/LucMiaz/PFASGroups/tree/v3.2.2 and 10.5281/zenodo.19393289. The Python module can be installed via PyPI (pip install PFASGroups, https://pypi.org/project/PFASGroups/) and from source (see GitHub repository for instructions). The web application is accessible on https://chem.cogitopia.dev/. The data used in this study are openly accessible from the cited references, and the generated results are available in the GitHub repository.

## Notes

Supporting details (PDF)
