# Supplementary material for: PFASGroups: An Open-Source Framework for Automated Identification, Structural Classification, and Prioritization of Per- and Polyfluoroalkyl Substances
Source: J Chem Inf Model. 2026 Jul 2;66(14):7813–20. doi: 10.1021/acs.jcim.6c01058 (PMC13417881; doi:10.1021/acs.jcim.6c01058)
Supplement: Supplementary file 1 [file ci6c01058_si_001.pdf]

Supplementary Information for

PFASGroups: An Open-Source Framework for Automated Identification, Structural Classification, and Prioritization of Per- and Polyfluoroalkyl Substances

Luc T. Miaz, Ian T. Cousins, Ida Rahu**

Department of Environmental Science, Stockholm University, Svante Arrhenius Väg 8, SE-106 91 Stockholm, Sweden
* Corresponding authors: L.T.M – luc.miaz@aces.su.se; I.R – ida.rahu@aces.su.se

Contents

Introduction S3
S1. Details on HalogenGroups S4
S1.1. Architecture S4
S1.2. Configuration parameters S4
S1.3. Default component names per halogen S4
S2. Component SMARTS patterns S5
S2.1. Fluorine-based component SMARTS S5
S2.2. Halogen-generalized SMARTS S6
S3. Definitions of PFAS groups S7
S3.1. Origin of the three group families S7
S3.2. Group definition fields S8
S4. Component metrics S20
S4.1. Basic graph metrics S20
S4.2. Computational implementation S26
S4.3. PFASEmbedding and PFASEmbeddingSet S27
S5. PFAS regulatory definitions S30
S5.1. OECD definition S30
S5.2. EU Restriction Proposal S31
S5.3. UK Regulatory Management Option Analysis (RMOA) S31
S5.4. EPA's Office of Pollution Prevention and Toxics (OPPT) definition S31
S5.5. PFASSTRUCTv5 .... S32
S6. Molecule prioritization S34
S6.1. Reference-based prioritization S34

S6.2. Intrinsic prioritization S34
S7. Software environment S35
S8. Benchmarking and validation S36
S8.1. Computational complexity S36
S8.2. Comparison with PFAS-Atlas S38
S8.3. PubChem fluorotelomer validation S42
S8.4. Validation of PFAS definitions S43
S8.5. Fingerprint benchmarking on ToxCast endpoints S43
References S50

Introduction

This supplementary document provides comprehensive details on the halogen group classification system implemented in `PFASGroups`/`HalogenGroups`, with primary focus on per- and polyfluoroalkyl substances (PFAS). Section S1 introduces the `HalogenGroups` module, an extension of `PFASGroups` to all halogen families. Section S2 documents the SMARTS patterns used to identify fluorinated and halogenated components in molecular graphs. Section S3 presents the full group classification table, including SMARTS patterns, formula constraints, and component constraints. Section S4 defines all per-group and molecule-wide metrics, their implementation, and their use in fingerprints/embeddings. Section S5 documents five major PFAS regulatory definitions used by default in the module, along with their structural criteria. Section S6 describes the built-in molecule prioritization module and its two scoring strategies. Section S7 lists the software environment and package versions. Section S8 presents accuracy and performance benchmarks, including `PFAS-Atlas` comparison and timing analysis, and predictive performance benchmarks on ToxCast toxicological endpoints against `ToxPrint` and `TxP_PFAS` fingerprints.

S1. Details on HalogenGroups

HalogenGroups is the multi-halogen extension of PFASGroups. Whereas PFASGroups operates exclusively on fluorinated substructures, HalogenGroups applies the same SMARTS-based group-detection and graph-metric pipeline to all four halogens, fluorine (F), chlorine (Cl), bromine (Br), and iodine (I), or to any user-specified subset. The module is activated by specifying `halogens='F'` (default, equivalent to PFASGroups) or a list such as `halogens = ['F', 'Cl', 'Br', 'I']` during parsing.

S1.1. Architecture

HalogenGroups operates by performing independent halogen-specific parsing steps, followed by consolidation into a unified output representation. For each selected halogen $h \in \{\mathrm{F}, \mathrm{Cl}, \mathrm{Br}, \mathrm{I}\}$, the algorithm executes a workflow analogous to PFASGroups, with all detection, filtering, and metric calculations restricted to the specified halogen.

S1.2. Configuration parameters

- **`halogens`**: Halogen(s) to analyze. Accepts a single symbol ('F') or a list (['F', 'Cl', 'Br', 'I']). Defaults to 'F' in PFASGroups and to all four halogens in HalogenGroups.
- **`saturation`**: Component saturation requirement. 'per' restricts detection to fully halogenated sp^3 chains (e.g., perfluoroalkyl), 'poly' captures partially halogenated chains, and 'both' (default) includes all components.
- **`form`**: Structural form filter. 'alkyl' (default) targets aliphatic halogenated chains, 'cyclic' targets saturated ring systems, and 'aryl' targets aromatic ring systems with halogenated substituents or halogenated aromatic carbons.
- **`group_selection`**: Controls which of the 119 PFAS groups are evaluated. For embedding generation, the aggregated telomer group and the selected halogen group (e.g., the fluorine group when halogens='F') are omitted because their information is already captured by more specific group assignments, thereby reducing feature collinearity. As a result, the default fluorine-based embedding contains 117 features. Presets include 'all', 'oecd', 'generic', 'telomers', and 'generic+telomers' (see Section S4.3).

S1.3. Default component names per halogen

Table S1 lists the standard component names assigned for each halogen and saturation category. These names are used in the `ComponentModel.componentType` field of the output and in the group definition `componentConstraints`.

Table S1. Default halogenated component names by halogen and saturation type.

Halogen	Per-substituted	Poly-substituted
F	Perfluoroalkyl	Polyfluoroalkyl
Cl	Perchloroalkyl	Polychloroalkyl
Br	Perbromoalkyl	Polybromoalkyl
I	Periodoalkyl	Polyiodoalkyl

S2. Component SMARTS patterns

The component identification step uses a library of SMARTS patterns stored in `PFASGroups/data/component_smarts_halogens.json`. Each pattern targets a specific combination of halogen, saturation state, and structural form, and is used to seed the connected-component search that extracts fluorinated (or more generally halogenated) carbon substructures from the molecular graph.

S2.1. Fluorine-based component SMARTS

The fluorine patterns are the most extensively developed and serve as the reference design for all other halogens.

Perfluoroalkyl

The *Perfluoroalkyl* component captures fully fluorinated sp^3 carbon chains.

SMARTS:

```
[C$([#6H0X4]([F,$([CX4H0]F),$([CX4H0][CX4H0]F),$([#8][CX4H0]F)])([!#17
,!#35,!#54])([!#17,!#35,!#54])[CX4H0,F])!R,C$([#6H0X4]([#6H0X4])([#6H0
X4])[#6H0X4])!R,O$([#8]([CX4H0](F)F)[CX4H0](F)F)]
```

This pattern matches:

- **CF_2/CF_3 chain carbons** (`[#6H0X4](…)[CX4H0,F]`): acyclic, hydrogen-free, tetravalent carbons bonded to at least one F atom (directly or via CF-containing fragments, including ether linkages), and to two substituents that are not Cl, Br, or I.
- **Quaternary perfluoroalkyl junctions** (`[#6H0X4]([#6H0X4])([#6H0X4])[#6H0X4]`): fully substituted branching points within perfluoroalkyl backbones.
- **Perfluoroether oxygen bridges** (`O([…](F)F)[…](F)F`): ether oxygens flanked by CF_2 units on both sides, enabling correct identification of perfluoropolyether (PFPE) backbones.

Polyfluoroalkyl

The *Polyfluoroalkyl* component captures partially fluorinated sp^3 carbons, defined as chains containing at least one C–F bond in combination with C–H, C–Cl, C–Br, or C–I bonds.

SMARTS:

```
[C$([CX4]([#9,$([#6X4][#9]),$([#8]CF)])[C,F,#1,#17,#35,#53])!R,C$([#6X
4]([#6X4])([#6X4])[#6X4])!R,C$([#6X4]([#6X4])[#6X4]F)!R,O$([#8](CF)[C$
(C[F,C$(CC(F))])])]
```

The pattern matches sp^3 carbons bonded to a fluorine atom (directly or via a CF-bearing carbon), allowing connections to H, Cl, Br, and I. Ether oxygen bridges adjacent to CF_2 are also captured for polyfluoroether detection.

Cyclic and aryl variants

Cyclic (form='cyclic') and aryl (form='aryl') patterns are derived from the alkyl definitions with appropriate modifications. Cyclic patterns incorporate the ring-membership flag (R), while aryl patterns replace aliphatic carbon descriptors (CX4) with aromatic carbon descriptors (c). This enables the detection of perhalogenated alicyclic systems (e.g., chlordane- or HBCD-like structures) as well as halogenated aromatic compounds.

S2.2. Halogen-generalized SMARTS

HalogenGroups supports the same component definitions described above, with fluorine (F or #9) systematically replaced by the corresponding halogen atomic number (Cl #17, Br #35, I #53). This generalization preserves the structural logic of the fluorine-based patterns while extending applicability across all supported halogens.

S3. Definitions of PFAS groups

The default classification system comprises 119 groups in total and is applicable across all supported halogens (F, Cl, Br, and I), with PFAS-related groups as the primary focus. These groups are organized into three families:

- 28 OECD- defined groups (IDs 1 to 28)
- 48 generic functional groups (IDs 29 to 76)
- 43 fluorotelomer-specific groups (IDs 77 to 119)

S3.1. Origin of the three group families

OECD-defined groups (IDs 1–28). The 28 OECD chemical classes implemented here were derived from the compound families illustrated in Figure 9 of the OECD report "Reconciling Terminology of the Universe of Per- and Polyfluoroalkyl Substances: Recommendations and Practical Guidance"[1]. Each group was translated into a combination of SMARTS substructure patterns, molecular formula constraints (such as atom counts and F/C ratio thresholds), and component-type requirements (such as *Perfluoroalkyl* or *Polyfluoroalkyl*). This enabled the OECD structural definitions to be operationalized within a cheminformatics workflow. The resulting classification scheme was iteratively validated against the OECD 2018 PFAS list[2], which provided a ground-truth set of 3,414 known PFAS used to identify and correct systematic misclassifications.

Generic functional groups (IDs 29–76). The generic groups originated from two complementary sources. First, each OECD chemical class implies a broader functional analog. For example, the OECD class perfluoroalkyl carboxylic acids implies the generic carboxylic acid motif, and the OECD class sulfonyl fluorides implies the generic sulfonyl halide motif. These generic counterparts were derived by relaxing the stricter formula and component constraints of the OECD groups while retaining the core SMARTS patterns. Second, additional functional groups were introduced by systematically examining residual unclassified compounds from the OECD PFAS list and identifying recurring structural motifs not captured by the OECD scheme, such as rare heterocycles, coupling agents, and surface-treatment reagents.

Fluorotelomer-specific groups (ID 77–119). The telomer group library was developed iteratively using a compound set retrieved from PubChem using the keyword search "fluorotelomer" (785 compounds). This dataset spans a broad range of commercially relevant fluorotelomer derivatives, including alcohols, carboxylic acids, sulfonic acids, phosphates, acrylates, and betaines, and was used to identify functional head groups absent from the initial OECD-derived library. For each telomer class that was well represented in the PubChem set, a new group was added with a `linker_smarts` requirement specifying that only CH_2 atoms, or CH_2 and O atoms in ethoxylate variants, may connect the perfluorinated tail to the functional head group. This CH_2-based linker constraint is the key structural feature that distinguishes fluorotelomers from directly attached OECD perfluoroalkyl classes and enables both classes to be detected simultaneously in multifunctional molecules.

S3.2. Group definition fields

Each entry in the group library is defined by the fields below, which together specify its structural pattern, constraints, and validation set.

- `id` **(integer)**: Unique integer identifier. IDs are stable across releases and are used to reference groups in results, fingerprint vectors, and Table S2.
- `name` **(string)**: Human-readable label describing the chemical class, for example, "OECD perfluoroalkyl carboxylic acids". The name appears directly in parsed output records.
- `smarts` **(SMARTS patterns)**: A mapping of one or more SMARTS substructure patterns to the minimum number of non-overlapping matches required in the molecule. When this field is null and shown as a dash in the table, the group is identified solely by component type, without an explicit substructure query. If multiple SMARTS patterns are listed, each must satisfy its own minimum match requirement.
- `constraints` **(formula constraints)**: A set of molecular formula conditions applied to the whole molecule (represented in the Table S2 as the *Formula Constraints* column). Supported condition types include only, meaning that the molecule may contain only the listed elements; `eq`, `gte`, `lte`, indicating exact, minimum, and maximum counts for a given element; and `rel`, indicating a relational inequality between element counts, for example $n_{\mathrm{C}} \cdot 2 \geq n_{\mathrm{F}} + 0.5$).
- `test` **(examples and counter-examples)**: An optional validation dictionary keyed by halogen symbol (F, Cl, Br, I). Each entry contains a list of examples, that is, SMILES strings that *must* match the group, and counter-examples, that is, SMILES strings that *must not* match. These are executed automatically by the unit-test suite to guard against regressions.

Component constraints

Each group definition may also include optional parameters specifying which type of fluorinated (or halogenated) component is required, how far a functional-group SMARTS match may lie from that component, and which atoms are permitted to bridge the gap. These parameters are summarized in the *Component Constraints* column of Table S2 using the abbreviations defined below.

- `componentSmarts` **(component type, abbreviated "*Comp.*")**: Specifies the required pre-defined fluorinated or halogenated component by name, for example, *Perfluoroalkyl* or *Polyfluoroalkyl*. When no named component is given explicitly, the component type is inferred from the combination of `componentSaturation`, `componentForm`, and `componentHalogens`.
- `componentSaturation` **(saturation, abbreviated "*Sat.*")**: Specifies whether the component must be perhalogenated (`per`), meaning that every non-hydrogen position is occupied by the target halogen, or polyhalogenated (`poly`), meaning that at least one but not all positions are halogen-substituted. When this field is `null` or `both`, either saturation state is accepted.

- **componentForm (structural form, abbreviated "*Form*")**: Restricts the topology of the required component to alkyl (acyclic carbon chain, default), cyclic (non-aromatic ring), or aryl (aromatic ring).
- **excludeHalogens (exclusion list, abbreviated "*Excl.*")**: Lists the halogen(s) for which a group is *excluded* during parsing. This prevents double-counting when a more specific group already covers the same structural motif. For example, the generic fluoride group (ID 48) carries Excl.: F because fluorinated compounds are already covered by the OECD and telomer hierarchies.
- **max_dist_from_comp (maximum distance from component)**: By default, max_dist_from_comp = 0, meaning that every atom matched by the functional-group SMARTS must either belong to the halogenated component itself or be directly bonded to one of its atoms, corresponding to a graph distance of at most 1. Setting max_dist_from_comp = d, where $d > 0$, relaxes this requirement: the algorithm accepts a SMARTS match if at least one matched atom lies within *d* bonds of the nearest component atom. This parameter, therefore, controls the maximum permitted length of the non-halogenated bridge between the functional head group and the halogenated tail. For example, OECD perfluoroalkyl dicarboxylic acids (ID 8) use max_dist_from_comp = 1 because the two carboxylate groups flank the perfluorinated chain and each lies one bond outside it, whereas telomer groups use values of up to 16 to accommodate variable-length CH_2 spacers.
- **linker_smarts (linker atom filter)**: When max_dist_from_comp > 0, atoms along every shortest path from the halogenated component to the matched functional-group atom are checked against the linker_smarts pattern. A match is accepted only if *every* intermediate atom on at least one such path satisfies this pattern. When linker_smarts is null or None (default), no restriction is imposed on linker atoms. For telomers, the linker SMARTS is typically [CH2X4], corresponding to an sp^3 methylene group, meaning that only CH_2 carbons may bridge the perfluorinated tail and the functional head group. The ethoxylate variant (ID 84) extends this to [CH2X4,O$(O([CH2])[CH2])] to also allow ether oxygens within the spacer. This constraint gives telomer groups their structural specificity: a perfluoroalkyl carboxylic acid, such as PFOA, which is directly attached, and a fluorotelomer carboxylic acid, such as 6:2 FTCA, which is CH_2-linked, share the same carboxylate SMARTS pattern but are distinguished by whether the path to the perfluorinated component passes exclusively through CH_2 carbon atoms. The linker length, expressed as the number of CH_2 C atoms, is stored as the n_spacer per-component metric (Section S4).

Component-type atom constraints

In addition to the group-level parameters above, each component type definition declared in component_smarts_halogens.json may include a constraints entry evaluated against the full atom count of the matched component, including backbone carbons and all directly attached halogen atoms. Supported keys are gte, indicating a minimum element count (e.g., "hal" ≥ 2), and exclude, indicating a list of forbidden elements. Unlike the group-level parameters, these constraints are attached to the component type itself and therefore apply uniformly to every group requiring that

component type. The built-in polyhalogenated alkyl component types, namely *Polyfluoroalkyl*, *Polychloroalkyl*, *Polybromoalkyl*, and *Polyiodoalkyl*, each include the condition "`hal`" ≥ `2` to ensure that components classified as polyhalogenated bear at least two halogen substituents. In Table S2, this is shown in the *Component Constraints* column.

In Table S2, the six group-level parameters are abbreviated in the *Component Constraints* column as "`Comp.: type`", "`Sat.: per/poly`", "`Form: form`", "`Excl.: halogen`", "`Max dist.: d`", and "`Linker: pattern`", respectively. Groups without a linker entry use `max_dist_from_comp = 0` and therefore impose no linker restriction.

Table S2 presents all groups included in the classification algorithm together with their SMARTS patterns, formula constraints, and component constraints.

Table S2. Groups used in the classification algorithm, including their SMARTS patterns, formula constraints, and component constraints

Category	ID	Group name	SMARTS patterns	Formula constraints	Component constraints
**OECD**	1	OECD perfluoroalkyl alcohols	[#6X4$([#6!$([#6]=O)][OH1,Oh1,O-])]	gte(F≥2); only(C,F,O,H); rel(C≤(F+O-1)/2)	component=Perfluoroalkyl
**OECD**	2	OECD telomer alcohols	[C$([#6][C$([CH2][OH1,Oh1,O-]),C$([CH2][CH2][OH1,Oh1,O-]),C$([CH2][CH2][CH2][OH1,Oh1,O-]),C$([CH2][CH2][CH2][CH2][OH1,Oh1,O-]),C$([CH2][CH2][CH2][CH2][CH2][OH1,Oh1,O-]),C$([CH2][CH2][CH2][CH2][CH2][CH2][OH1,Oh1,O-]),C$([CH2][CH2][CH2][CH2][CH2][CH2][CH2][OH1,Oh1,O-]),C$([CH2][CH2][CH2][CH2][CH2][CH2][CH2][CH2][OH1,Oh1,O-]),C$([CH2][CH2][CH2][CH2][CH2][CH2][CH2][CH2][CH2][OH1,Oh1,O-]),C$([CH2][CH2][CH2][CH2][CH2][CH2][CH2][CH2][CH2][CH2][OH1,Oh1,O-])])]|C(F)(F)F	eq(O=1); gte(F≥2)	component=Perfluoroalkyl; max_dist=0
**OECD**	3	OECD perfluoroalkanes		gte(F≥2); only(C,F,H); rel(C≤(F-1)/2)	component=Perfluoroalkyl
**OECD**	4	OECD semi-fluorinated alkanes	[#6X4$([#6](F)(F))]\|[#6X4$([#6][H,I,Br,Cl])]	eq(O=0, S=0); gte(F≥2); rel(C≤(Cl+I+Br+F+H-1)/2)	component=Polyfluoroalkyl
**OECD**	5	OECD perfluoroalkenes	[#6$([#6H0](F)=[#6H0]F)]	gte(F≥2); only(C,F,H); rel(C≤(F+0)/2)	max_dist=16
**OECD**	6	OECD perfluoroalkyl carboxylic acids	[#6$([#6][#6](=O)([OH1,Oh1,O-]))]	eq(O=2); gte(F≥2); only(C,F,O,H); rel(C≤(F+0.5)/2)	component=Perfluoroalkyl
**OECD**	7	OECD polyfluoroalkyl carboxylic acids	[#6$([#6][#6](=O)([OH1,Oh1,O-]))]	eq(O=2); gte(F≥2); only(C,F,O,H); rel(C≤(F+H+Cl+Br+I+0)/2)	component=Polyfluoroalkyl

<table>
<tr><td>OECD</td><td>8</td><td>OECD perfluoroalkyl dicarboxylic acids</td><td>[#6](=O)[OH1,Oh1,O-] ×2</td><td>eq(O=4);
gte(F≥2);
only(C,F,O,H);
rel(C≤(F+2)/2)</td><td>component=Perfluoroalkyl;
max_dist=1</td></tr>
<tr><td>OECD</td><td>9</td><td>OECD perfluoroalkyl ether carboxylic acids</td><td>[#6$([#6][#6](=O)([OH1,Oh1,O-]))]|[#6$([#6]([#8X2H0][#6!$(C=O)]))!$([#6](=O))]</td><td>gte(O≥3, F≥2);
only(C,F,O,H);
rel(C≤(F+0.5)/2)</td><td>component=Perfluoroalkyl</td></tr>
<tr><td>OECD</td><td>10</td><td>OECD polyfluoroalkyl ether carboxylic acids</td><td>[#6$([#6][#6](=O)([OH1,Oh1,O-]))]|[#6$([#6]([#8X2H0][#6!$(C=O)]))!$([#6](=O))]</td><td>gte(O≥3, F≥2);
only(C,F,H,Cl,Br,I,O);
rel(C≤(F+H+Cl+Br+I+0)/2)</td><td>component=Polyfluoroalkyl</td></tr>
<tr><td>OECD</td><td>11</td><td>OECD hydrofluoroethers</td><td>[#6][#8][#6]</td><td>gte(O≥1, F≥2);
only(C,F,O,H);
rel(C≤(F+H-1)/2)</td><td>component=Polyfluoroalkyl</td></tr>
<tr><td>OECD</td><td>12</td><td>OECD hydrofluorocarbons</td><td></td><td>gte(F≥2);
only(C,F,H);
rel(C≤(F+H-1)/2)</td><td>component=Polyfluoroalkyl</td></tr>
<tr><td>OECD</td><td>13</td><td>OECD perfluoroalkyl iodides</td><td></td><td>gte(I≥1, F≥2);
rel(C≤(F+I-1)/2)</td><td>component=Perfluoroalkyl</td></tr>
<tr><td>OECD</td><td>14</td><td>OECD perfluoroalkyl ketones</td><td>[#6X4$([#6]C(=O)[#6])]</td><td>gte(F≥2);
only(C,F,O);
rel(C≤(F+0)/2)</td><td>component=Perfluoroalkyl</td></tr>
<tr><td>OECD</td><td>15</td><td>OECD semi-fluorinated alkyl ketones</td><td>[#6X4$([#6]C(=O)[#6])]</td><td>gte(O≥1, F≥3);
rel(C≤(Cl+I+Br+F+H-1)/2)</td><td>component=Polyfluoroalkyl</td></tr>
<tr><td>OECD</td><td>16</td><td>OECD hydrofluoroolefins</td><td>[C$(C=C)]</td><td>gte(F≥2);
only(C,F,H);
rel(C≤(F+H+0)/2)</td><td>component=Polyfluoroalkyl;
max_dist=16;
linker=[CH2]</td></tr>
<tr><td>OECD</td><td>17</td><td>OECD perfluoropolyethers</td><td>[#6$([#6]O[#6])]</td><td>gte(O≥2, F≥2);
only(C,F,O);
rel(C≤(F-1)/2)</td><td>component=Perfluoroalkyl</td></tr>
<tr><td>OECD</td><td>18</td><td>OECD side-chain fluorinated aromatics</td><td>[#6X4$([#6]a)]</td><td>gte(F≥2)</td><td>component=Perfluoroalkyl</td></tr>
<tr><td>OECD</td><td>19</td><td>OECD perfluoroalkyl ether sulfonic acids</td><td>[#6$([#6][#16](=O)(=O)([OH]))]|
[#6$([#6]([#8X2H0][#6!$(C=O)]))!$([#6](=O))]</td><td>eq(S=1);
gte(O≥4, F≥2);
only(C,F,H,S,O);
rel(C≤(F-0.5)/2)</td><td>component=Perfluoroalkyl</td></tr>
<tr><td>OECD</td><td>20</td><td>OECD polyfluoroalkyl ether sulfonic acids</td><td>[#6$([#6][#16](=O)(=O)([OH]))]|
[#6$([#6]([#8X2H0][#6!$(C=O)]))!$([#6](=O))]</td><td>eq(S=1);
gte(O≥4, F≥2);
only(C,F,H,Cl,Br,I,S,O);
rel(C≤(F+H+Cl+Br+I-1)/2)</td><td>component=Polyfluoroalkyl</td></tr>
</table>

**OECD**	21	OECD perfluoroalkyl sulfinic acids	[#6$([#6][#16X3](=O)[OH1,Oh1,O-])]	eq(S=1);<br>gte(F≥2);<br>only(C,F,S,O,H);<br>rel(C≤(F-0.5)/2)	component=Perfluoroalkyl
**OECD**	22	OECD perfluoroalkyl sulfonic acids	[#6$([#6][#16](=O)(=O)[OH1,Oh1,O-])]	eq(S=1, O=3);<br>gte(F≥2);<br>only(C,F,H,S,O);<br>rel(C≤(F-0.5)/2)	component=Perfluoroalkyl
**OECD**	23	OECD polyfluoroalkyl sulfonic acids	[#6$([#6][#16](=O)(=O)[OH1,Oh1,O-])]	eq(S=1, O=3);<br>gte(F≥2);<br>only(C,F,H,Cl,Br,I,S,O);<br>rel(C≤(F+H+Cl+Br+I-1)/2)	component=Polyfluoroalkyl
**OECD**	24	OECD perfluoroalkyl disulfonic acids	[#16](=O)(=O)[OH1,Oh1,O-] ×2	eq(S=2, O=6);<br>gte(F≥2);<br>only(C,F,H,Cl,Br,I,S,O);<br>rel(C≤(F+0)/2)	component=Perfluoroalkyl;<br>max_dist=1
**OECD**	25	OECD perfluoroalkyl sulfonyl fluorides	[#6$([#6][#16](=O)(=O)[F])]	gte(F≥2);<br>rel(C≤(F-1)/2)	component=Perfluoroalkyl;<br>max_dist=0
**OECD**	26	OECD perfluoroalkyl phosphinic acids	[#6X4$([#6][#15](=[#8])([#8])[#1,#6])]	eq(P=1, O=2);<br>gte(F≥2);<br>only(C,F,P,O,H);<br>rel(C≤(F-0.5)/2)	component=Perfluoroalkyl
**OECD**	27	OECD perfluoroalkyl phosphonic acids	[#6$([#6][#15](=[#8])([#8])[#8])]	eq(P=1, O=3);<br>gte(F≥2);<br>only(C,F,P,O,H);<br>rel(C≤(F-0.5)/2)	component=Perfluoroalkyl
**OECD**	28	OECD perfluoroalkyl tertiary amines	[#6$([#6][#7D3])]	gte(F≥2);<br>only(C,F,H,N);<br>rel(C≤(F-1.5)/2)	component=Perfluoroalkyl
**generic**	29	acrylates	[CH2$(COC(=O)[CH1]=[CH2])]	gte(O≥2)	max_dist=0
**generic**	30	acyl halides	[#6$([#6][#6](=O)[#9,#17,#35,#53])]		max_dist=0
**generic**	31	alcohols	[#6$([#6!$([#6]=O)][OH1,Oh1,O-])]	gte(O≥1)	max_dist=0
**generic**	32	aldehydes	[C$(C[CH1,C$(C(F))](=O))]	gte(O≥1)	
**generic**	33	alkenes	[#6$(C[#6]=[#6])]		max_dist=0
**generic**	34	perhalogenated alkyl compounds			saturation=per

**generic**	35	polyhalogenated alkyl compounds			saturation=poly
**generic**	36	alkynes	[#6$([#6]#[#6])]		max_dist=1
**generic**	37	perhalogenated aryl compounds			saturation=per; form=aryl
**generic**	38	polyhalogenated aryl compounds			saturation=poly; form=aryl
**generic**	39	benzodioxoles	[C$(C[c$(c1ccc2OC(F)(F)Oc2c1),c$(c1cc2OC(F)(F)Oc2cc1),c$(c1c2OC(F)(F)Oc2ccc1)]),C$(C(F)(F)(Oc2c1)Oc2ccc1)]	gte(O≥2)	max_dist=1
**generic**	40	benzoyl peroxides	[#6X4$([#6][#6](=[#8])[#8][#8][#6](=[#8]))]		
**generic**	41	bromides	[#6$([#6][#35])]	gte(Br≥1)	max_dist=1
**generic**	42	carboxylic acids	[$([#6][#6](=O)[OH1,Oh1,O-])]	gte(O≥2)	max_dist=0
**generic**	43	chlorides	[#6$([#6][#17])]	gte(Cl≥1)	max_dist=1
**generic**	44	perhalogenated cyclic compounds			saturation=per; form=cyclic
**generic**	45	polyhalogenated cyclic compounds			saturation=poly; form=cyclic
**generic**	46	esters	[#6X4$([#6](=O)O[#6]),#6X4$([#6]O[#6](=O)[#6])]	gte(O≥2)	max_dist=0
**generic**	47	ethers	[#6$([#6]([#8X2H0][#6!$(C=O)]))!$([#6](=O))]	gte(O≥1)	max_dist=0
**generic**	48	fluorides	[#6$([#6][#9])]	gte(F≥1)	max_dist=1
**generic**	49	glucuronates	[CX4$(C[O$(O[C@@H]1O[C@H](C(=O)O)[C@@H](O)[C@H](O)[C@H]1O),O$(O[C@H]1[C@H](O)O[C@H](C(=O)O)[C@@H](O)[C@@H]1O),O$(O[C@@H]1[C@@H](O)[C@H](O)O[C@H](C(=O)O)[C@H]1O),O$(O[C@H]1[C@H](O)[C@@H](O)[C@H](O)O[C@@H]1C(=O)O)])]	gte(O≥7)	max_dist=0
**generic**	50	iodides	[#6$([#6][#53])]	gte(I≥1)	max_dist=1
**generic**	51	ketones	[#6$([#6]C(=O)[#6])]	gte(O≥1)	
**generic**	52	methacrylates	[CH2$(COC(=O)C(C)=[CH2])]	gte(O≥2)	max_dist=0
**generic**	53	peroxides	[#6X4$([#6][#8][#8])]	gte(O≥2)	
**generic**	54	side-chain aromatic compounds	[#6X4$([#6]a)]		

**generic**	55	sulfenic acids	[#6$([#6][#16X2][OH1,Oh1,O-])]	gte(S≥1, O≥1)	max_dist=0
**generic**	56	sulfenyl halides	[#6$([#6][#16X2][F,Cl,Br,I])]	gte(S≥1, O≥2)	max_dist=0
**generic**	57	sulfinic acids	[#6$([#6][#16X3](=O)[O])]	gte(S≥1, O≥2)	max_dist=0
**generic**	58	sulfinyl amido sulfonic acids	[C$(CS(=O)[CH2][CH2]C(=O)[NH]C([CH3])([CH3])[CH2]S(=O)(=O)[OH1,Oh1,O-])]	gte(O≥5, S≥2, N≥1)	max_dist=0
**generic**	59	sulfonamides	[CX4$([#6][#16]([#7])(=[#8])=[#8]),CX4$([#6][#7][#16](=[#8])=[#8])]	gte(S≥1, N≥1, O≥2)	max_dist=0
**generic**	60	sulfonamidoethanols	[C$(CS(=O)(=O)NCC[OH1,Oh1,O-])]	gte(N≥1, O≥3, S≥1)	max_dist=0
**generic**	61	sulfonic acids	[#6$([#6][#16](=O)(=O)O)]	gte(S≥1, O≥3)	max_dist=0
**generic**	62	sulfonyl halides	[#6$([#6][#16](=O)(=O)[F,Cl,Br,I])]	gte(S≥1, O≥2)	max_dist=0
**generic**	63	sulfonyl propanoic acids	[C$(CS(=O)(=O)[CH2][CH2]C(=O)[OH1,Oh1,O-])]	gte(O≥4, S≥1)	max_dist=0
**generic**	64	sulfuric acids	[#6$([#6]O[#16](=O)(=O)O)]	gte(S≥1, O≥4)	max_dist=0
**generic**	65	thioester keto dicarboxylic acids	[C$(CC(=O)SC(=O)C([OH1,Oh1,O-])C(=O)[OH1,Oh1,O-])]	gte(O≥5, S≥1)	max_dist=0
**generic**	66	thiocyanates	[CX4$(CSC#N)]	gte(N≥1, S≥1)	max_dist=0
**generic**	67	phosphinic acids	[#6$([#6][#15](=[#8])([#8])[!$([#8])])]	gte(P≥1, O≥2)	max_dist=0
**generic**	68	phosphonic acids	[#6$([#6][#15]([#8])(=[#8])[#8])]	gte(P≥1, O≥3)	max_dist=0
**generic**	69	amides	[$([#6]([#7H0,#7H1,#7h1,#7H2])=[#8]),$([#6]~[#7H0,#7H1,#7h1,#7H2][#6X3]=[#8])]	gte(N≥1, O≥1)	max_dist=1
**generic**	70	amines	[#6$([#6!$([#6]=O)][N!$(*[#6]=O)])]	gte(N≥1)	
**generic**	71	heterocyclic azines	[$(*[#7r6,$([#6r6,#8r6,#16r6]:[#7r6]),$([#6r6,#8r6,#16r6]:[#6r6,#8r6,#16r6]:[#7r6]),$([#6r6,#8r6,#16r6]:[#6r6,#8r6,#16r6]:[#6r6,#8r6,#16r6]:[#7r6])])]	gte(N≥1)	max_dist=1

<table>
<tr><td>generic</td><td>72</td><td>heterocyclic azoles</td><td>[$(*[#7r5,$([#6r5,#8r5,#16r5]:[#7r5]),$([#6r5,#8r5,#16r5]:[#6r5,#8r5,#16r5]:[#7r5])])]</td><td>gte(N≥1)</td><td>max_dist=1</td></tr>
<tr><td>generic</td><td>73</td><td>betaines</td><td>[CX4$([#6]N(C)(C)CC(=O)[OH1,Oh1,O-])]</td><td>gte(N≥1, O≥2)</td><td>max_dist=0</td></tr>
<tr><td>generic</td><td>74</td><td>glycine derivatives</td><td>[C$(C[NH2+,N$([N+]([CH3])[CH3])]CC(=O)[OH1,Oh1,O-])]</td><td>gte(N≥1, O≥2)</td><td>max_dist=0</td></tr>
<tr><td>generic</td><td>75</td><td>trichlorosilanes</td><td>[C$(C[Si](Cl)(Cl)Cl)]</td><td>gte(Si≥1, Cl≥3)</td><td>max_dist=0</td></tr>
<tr><td>generic</td><td>76</td><td>silanes</td><td>[C$(C[SiH3])]</td><td>gte(Si≥1, H≥3)</td><td>max_dist=0</td></tr>
<tr><td>telomer</td><td>77</td><td>telomer acrylates</td><td>[C$(C[CH2]OC(=O)[CH1]=[CH2])]</td><td>gte(O≥2)</td><td>saturation=per;
max_dist=16;
linker=[CH2X4]</td></tr>
<tr><td>telomer</td><td>78</td><td>telomer alcohols</td><td>[C$(C[CH2][OH1,Oh1,O-])]</td><td>gte(O≥1)</td><td>saturation=per;
max_dist=16;
linker=[CH2X4]</td></tr>
<tr><td>telomer</td><td>79</td><td>telomer aldehydes</td><td>[C$(C[CH2][CH1](=O))]</td><td>gte(O≥1)</td><td>saturation=per;
max_dist=16;
linker=[CH2X4]</td></tr>
<tr><td>telomer</td><td>80</td><td>telomer alkenes</td><td>[C$([CH1$(C(=C)C),CH2]=[CH1])]</td><td></td><td>saturation=per;
max_dist=16;
linker=[CH2X4,CX3H1!$(CO)]</td></tr>
<tr><td>telomer</td><td>81</td><td>telomer carboxylic acids</td><td>[C$(C[CH2]C(=O)[OH1,Oh1,O-])]</td><td>gte(O≥2)</td><td>saturation=per;
max_dist=16;
linker=[CH2X4]</td></tr>
<tr><td>telomer</td><td>82</td><td>telomer esters</td><td>[C$(C[CH2]OC(=O)[CH2]),C$(C[CH2]C(=O)O[CH2X4])]</td><td>gte(O≥2)</td><td>saturation=per;
max_dist=16;
linker=[CH2X4]</td></tr>
<tr><td>telomer</td><td>83</td><td>telomer ether carboxylic acids</td><td>[$([CH2X4,OH0X2][CH2]C(=O)[OH1,Oh1,O-])]|[O$(O([C])[C])]</td><td>gte(O≥3)</td><td>saturation=per;
max_dist=16;
linker=[CH2X4,OH0X2]</td></tr>
<tr><td>telomer</td><td>84</td><td>telomer ethoxylates</td><td>[C$(C[CH2][OH1,Oh1,O-])]|[O$(O(C)C)]</td><td>gte(O≥1)</td><td>saturation=per;
max_dist=16;
linker=[CH2X4,O$(O([CH2])[CH2])]</td></tr>
<tr><td>telomer</td><td>85</td><td>telomer glucuronic acids</td><td>[C$(C[CH2][O$(O[C@@H]1O[C@H](C(=O)O)[C@@H](O)[C@H](O)[C@H]1O),O$(O[C@H]1[C@H](O)O[C@H](C(=O)O)[C@@H](O)[C@@H]1O),O$(O[C@@H]1[C@@H](O)[C@H](O)O[C@H](C(=O)O)[C@H]1O),O$(O[C@H]1</td><td>gte(O≥7)</td><td>saturation=per;
max_dist=16;
linker=[CH2X4]</td></tr>
</table>

			[C@H](O)[C@@H](O)[C@H](O)O[C@@H]1C(=O)O)])]		
**telomer**	86	telomer iodides	[C$(C[CH2]I)]	gte(I≥1)	saturation=per; max_dist=16; linker=[CH2X4]
**telomer**	87	telomer methacrylates	[C$(C[CH2]OC(=O)C(C)=[CH2])]	gte(O≥2, H≥2)	saturation=per; max_dist=16; linker=[CH2X4]
**telomer**	88	telomer alkyls	[C$(C[CH2][CH3,CH2!$(C([CH2])[CH2])])]		saturation=per; max_dist=16; linker=[CH2X4]
**telomer**	89	telomer unsaturated carboxylic acids	C(=O)[OH1,Oh1,O-]|[CH1]=[C]	gte(O≥2)	saturation=per; max_dist=16; linker=[CH2X4,CX3H1]
**telomer**	90	telomer unsaturated halides	[C$([CH2,CH1$(C=C)](~[CH2])[sI,F,Cl,Br])]		saturation=per; max_dist=16; linker=[CH2X4,CX3H1!$(CO)]
**telomer**	91	telomer unsaturated iodides	[C$(C~[CH2,CH1$(C=C)]I)]	gte(I≥1)	saturation=per; max_dist=16; linker=[CH2X4,CX3H1!$(CO)]
**telomer**	92	telomer thioester hydroxy dicarboxylic acids	[C$(C[CH2]C(=O)SC([OH1,Oh1,O-])C(=O)[OH1,Oh1,O-])]	gte(O≥4, S≥1)	saturation=per; max_dist=16; linker=[CH2X4]
**telomer**	93	telomer thioether hydroxy dicarboxylic acids	[C$(C[CH2]C(=O)SCC([OH1,Oh1,O-])C(=O)[OH1,Oh1,O-])]	gte(O≥4, S≥1)	saturation=per; max_dist=16; linker=[CH2X4]
**telomer**	94	telomer formaldehyde bisulfite adducts	[C$(C[CH2]C([OH1,Oh1,O-])S(=O)(=O)[OH1,Oh1,O-])]	gte(O≥4, S≥1)	saturation=per; max_dist=16; linker=[CH2X4]
**telomer**	95	telomer sulfenic acids	[C$(C[CH2][#16][OH1,Oh1,O-])]	gte(S≥1, O≥1)	saturation=per; max_dist=16; linker=[CH2X4]
**telomer**	96	telomer sulfides	[C$(C[CH2][S!$(S(=O))!$(SO)])]	gte(S≥1)	saturation=per; max_dist=16; linker=[CH2X4]
**telomer**	97	telomer sulfinic acids	[C$(C[CH2][#16](=O)[OH1,Oh1,O-])]	gte(S≥1, O≥2)	saturation=per; max_dist=16; linker=[CH2X4]

**telomer**	98	telomer sulfinyl amido sulfonic acids	[C$(C[CH2]S(=O)[CH2][CH2]C(=O)[NH]C([CH3])([CH3])[CH2]S(=O)(=O)[OH1,Oh1,O-])]	gte(O≥5, S≥2, N≥1)	saturation=per; max_dist=16; linker=[CH2X4]
**telomer**	99	telomer sulfinyl compounds	[C$(C[CH2]S(=O)[#6!$(C~O)])]	gte(O≥1, S≥1)	saturation=per; max_dist=16; linker=[CH2X4]
**telomer**	100	telomer sulfonamides	[C$(C[CH2][#16]([#7])(=[#8])=[#8]),C$(C[CH2][#7][#16](=[#8])=[#8])]	gte(S≥1, N≥1, O≥2)	saturation=per; max_dist=16; linker=[CH2X4]
**telomer**	101	telomer sulfonamidoethanols	[C$(C[CH2]S(=O)(=O)NCC[OH1,Oh1,O-])]	gte(N≥1, O≥3, S≥1)	saturation=per; max_dist=16; linker=[CH2X4]
**telomer**	102	telomer sulfones	[C$(C[CH2][SX4](=O)(=O))]	gte(O≥2, S≥1)	saturation=per; max_dist=16; linker=[CH2X4]
**telomer**	103	telomer sulfonic acids	[C$(C[CH2][#16](=O)(=O)[OH1,Oh1,O-])]	gte(S≥1, O≥3)	saturation=per; max_dist=16; linker=[CH2X4]
**telomer**	104	telomer sulfonyl propanoic acids	[C$(C[CH2]S(=O)(=O)[CH2][CH2]C(=O)[OH1,Oh1,O-])]	gte(O≥4, S≥1)	saturation=per; max_dist=16; linker=[CH2X4]
**telomer**	105	telomer sulfuric acids	[C$(C[CH2]O[#16](=O)(=O)[OX2])]	gte(S≥1, O≥4)	saturation=per; max_dist=16; linker=[CH2X4]
**telomer**	106	telomer thioester keto dicarboxylic acids	[C$(C[CH2]C(=O)SC(=O)C([OH1,Oh1,O-])C(=O)[OH1,Oh1,O-])]	gte(O≥2)	saturation=per; max_dist=16; linker=[CH2X4]
**telomer**	107	telomer thiocyanates	[C$(C[CH2]SC#N)]	gte(N≥1, S≥1)	saturation=per; max_dist=16; linker=[CH2X4]
**telomer**	108	telomer monophosphates	[C$(C[CH2]OP(=O)([O!$(O[CH2])])[OH1,Oh1,O-])]	gte(O≥4)	saturation=per; max_dist=16; linker=[CH2X4]
**telomer**	109	telomer diphosphates	[C$(C[CH2]OP(=O)(O)O[CH2$(C[CH2,CX4$(C(F)(F)C)])])]	gte(O≥4)	saturation=per; max_dist=16; linker=[CH2X4]
**telomer**	110	telomer triphosphates	[C$(C[CH2]OP(=O)(O[CH2$(C[CH2,CX4$(C(F)(F)C)])])O[CH2$(C[CH2,CX4$(C(F)(F)C)])])]	gte(O≥4)	saturation=per; max_dist=16; linker=[CH2X4]

**telomer**	111	telomer tertiary amines	[C$(C[CH2]N([CH3])([CH3])C)]	gte(N≥1)	saturation=per; max_dist=16; linker=[CH2X4]
**telomer**	112	telomer amino propanoic acids	[C$(C[CH2]N([CH2$(C[CH2,C$(C(F)F)])])[CH2][CH2]C(=O)[OH1,Oh1,O-])]	gte(O≥2, N≥1)	saturation=per; max_dist=16; linker=[CH2X4]
**telomer**	113	telomer aminoethyl trimethylammonium compounds	[C$(C[CH2][CH2][NH,Nh,N-][CH2][CH2][N+]([CH3])([CH3])[CH3])]	gte(N≥2)	saturation=per; max_dist=16; linker=[CH2X4]
**telomer**	114	telomer betaines	[C$(C[CH2]N(C)(C)CC(=O)[OH1,Oh1,O-])]	gte(N≥1, O≥2)	saturation=per; max_dist=16; linker=[CH2X4]
**telomer**	115	telomer glycine derivatives	[C$(C[CH2][NH2+,N$([N+]([CH3])[CH3])]CC(=O)[OH1,Oh1,O-])]	gte(N≥1, O≥2)	saturation=per; max_dist=16; linker=[CH2X4,CH1X4$(C(F))]
**telomer**	116	telomer trichlorosilanes	[C$(C[CH2][Si](Cl)(Cl)Cl)]	gte(Si≥1, Cl≥3)	saturation=per; max_dist=6; linker=[CH2X4]
**telomer**	117	telomer silanes	[C$(C[SiH3])]	gte(Si≥1, H≥3)	saturation=per; max_dist=16; linker=[CH2X4]
**telomer**	118	telomer silyl compounds	[C$(C[CH2][Si](OC)(OC)OC)]	gte(Si≥1, O≥3)	saturation=per; max_dist=16; linker=[CH2X4]
**telomer**	119	telomer aggregates	Aggregated: returned if any telomer (IDs 77 to 118) is found		

S4. Component metrics

The `to_array()` method (Section S4.3) accepts a list of metric names via the `component_metrics` parameter. Each metric contributes a block of N_G columns, one per selected group, to the fingerprint matrix. For `group_selection='all'`, this corresponds to 117 groups in the default fluorine-only embedding, excluding the aggregated telomer group and the fluorine-only group. Optional molecule-level metrics may be appended as additional scalar features.

Table S3 defines all available metrics, including their mathematical formulations and chemical interpretation.

Aggregation

Per-group graph metrics are reduced to a single value per group using `aggregation='mean'` (default) or `'median'` when multiple components match the same group. Count-based metrics (`binary`, `count`, `max_component`, `total_component`) and all molecule-level metrics are not aggregated.

Graph representation

Molecules are represented as undirected graphs $G = (V, E)$ where vertices V correspond to atoms and edges E correspond to chemical bonds. The molecular graph is constructed using the `mol_to_nx` function, which converts *RDKit*[3] `Mol` objects to *NetworkX*[4] graph structures. For each fluorinated component $C \subseteq V$, a subgraph G_C is extracted for metric computation.

`HalogenGroups` computes graph-theoretical descriptors for each identified component to enable detailed structural characterization. The definitions and algorithms are implemented in `ComponentsSolverModel.py`.

S4.1. Basic graph metrics

For each matched functional group, distances are computed between SMARTS-matched atoms and structural graph features. These metrics capture the spatial positioning of functional groups relative to the halogenated component.

Component size

The component size $|C|$ is defined as the number of atoms in the halogenated carbon backbone, excluding attached hydrogen and halogen atoms. The method `get_full_component_atoms` expands components to include all directly bonded atoms (H, F, Cl, Br, I) for molecular mass calculations.

Branching index

The branching index B quantifies deviation from linearity:

$$B = 1 - \frac{2 \cdot \sum_j \max\big(0, \deg(v_j) - 2\big)}{\max(1, |C|)},$$

where $\deg(v_j)$ is the degree of the node j in a carbon-only subgraph C. The metric assesses deviation from linearity by counting each additional carbon-carbon edge beyond degree 2. The metric approaches 1 for linear chains and 0 for highly branched structures. Implemented in `calculate_branching`.

Spacer length

The spacer length m is defined as the number of linker atoms, typically C atoms from CH_2 units, on the shortest path between the perfluorinated component and the matched functional-group atom.

This corresponds to the m in the conventional "n:m fluorotelomer" nomenclature (e.g. $m = 2$ for a 4:2 FTOH, $m = 2$ for a 6:2 FTOH, $m = 2$ for a 6:2 FTCA). For non-telomer groups, $m = 0$ by convention.

Ring size

The ring size r is defined as:

$$r = \min\{|R|: R \in \mathcal{R}(G),\ R \cap C \neq \emptyset\}$$

where $\mathcal{R}(G)$ is the set of the smallest rings in a molecular graph G and C is the atom set of the matched component. For acyclic components, $r = 0$. For cyclic systems, r corresponds to the smallest overlapping ring (e.g., 6 for benzene, 5 for cyclopentane).

Eccentricity

For each node $v \in G_C$, the eccentricity $\epsilon(v)$ is:

$$\epsilon(v) = \max_{u \in G_C} d(v, u),$$

where $d(v, u)$ is the shortest-path distance computed via breadth-first search (BFS) using *NetworkX*'s `single_source_shortest_path_length`. Eccentricity measures how far a node is from the farthest point in the component.

Diameter and radius

Component-level metrics derived from eccentricity:

$$\text{Diameter:} \quad D(G_C) = \max_{v \in G_C} \epsilon(v)$$

$$\text{Radius:} \quad R(G_C) = \min_{v \in G_C} \epsilon(v)$$

The diameter represents the maximum separation between any two atoms, while the radius identifies the minimum eccentricity achievable. These metrics are computed using *NetworkX* functions `nx.diameter` and `nx.radius`.

Center

The center $Z(G_C)$ consists of nodes with minimum eccentricity:

$$Z(G_C) = \{v \in G_C : \epsilon(v) = R(G_C)\}$$

Nodes in the center minimize the maximum distance to all other nodes. Computed via `nx.center`.

Periphery

The periphery $P(G_C)$ consists of nodes with maximum eccentricity:

$$P(G_C) = \{v \in G_C : \epsilon(v) = D(G_C)\}$$

Periphery nodes are maximally distant from the component's interior. Computed via `nx.periphery`.

Barycenter

The barycenter $B(G_C)$ consists of nodes minimizing the total distance to all other nodes:

$$B(G_C) = \left\{ v \in G_C : \sum_{u \in G_C} d(v,u) = \min_{w \in G_C} \sum_{u \in G_C} d(w,u) \right\}$$

Unlike the center (which minimizes *maximum* distance), the barycenter minimizes *total* distance. Computed via `nx.barycenter`.

Effective graph resistance

Graph resistance R_{eff} quantifies the overall connectivity and redundancy of paths in the molecular graph. Following Ellens et al.[5] and *Networkx*'s implementation[6], the effective graph resistance is computed as

$$R_{eff} = N \sum_{i=1}^{N} \frac{1}{\mu_i}$$

where N is the number of vertices, $\{\mu_i\}_{i=1}^{N}$ are the eigenvalues of the Laplacian $Q = \Delta - A$ with Δ, the degree matrix and A, the adjacency matrix.

Computation control: The `limit_effective_graph_resistance` parameter enables selective computation:

- `None`: compute for all components
- integer > 0: compute only if $|C| <$ limit
- `0`: skip computation

Benchmarking (Section S8) shows that this contributes ~23% of the quadratic time coefficient.

Table S3. Available component and molecule-level fingerprint metrics, including formulas and structural interpretation. The "Agg." column indicates whether per-component values are aggregated when a group has multiple matched components.

Scope	Option	Bits	Type	Formula	Agg.	Structural insight
per group	`binary`	N_G	binary	$b_g = \mathbf{1}[\exists\, C_i \text{ matching } g]$	—	Presence/absence. Most information-dense per bit; default. Sufficient for OECD category assignment.
per group	`count`	N_G	int≥0	$c_g = \lvert\{C_i : C_i \text{ matches } g\}\rvert$	—	Structural repetition count. Distinguishes monomeric from oligomeric/polymeric structures.
per group	`max_component`	N_G	int≥0	$x_g = \max_i \lvert C_i \rvert$	—	C-atom count of the dominant fragment. Proxy for the longest chain; correlated with persistence and bioaccumulation.
per group	`total_component`	N_G	int≥0	$x_g = \sum_i \lvert C_i \rvert$	—	Total halogenated carbon burden. Measures cumulative fluorination load; useful for hazard scoring.
per group	`size`	N_G	int≥0	$s = \lvert V \rvert$	yes	Raw fragment size. Redundant with `max_component` for single-component matches; distinct for multiple components.
per group	`n_spacer`	N_G	int≥0	$n = \lvert\{v \in \text{linker}\}\rvert$	yes	Telomer CH_2 spacer length ("m" in "m:n" telomer notation). Zero for non-telomers; distinguishes 2:1 from 4:2 and 6:2 fluorotelomers.
per group	`ring_size`	N_G	int≥0	$r = \min\{\lvert R \rvert : R \in \mathcal{R}(G),\ R \cap C \neq \emptyset\}$	yes	Smallest ring overlapping the matched component. Zero for acyclic chains; 5 for azoles/furans; 6 for benzene and cyclohexane derivatives.
per group	`branching`	N_G	float [0,1]	$\beta = \frac{d(G)}{(\lvert V \rvert - 1)}$; $\beta = 1$ linear, $\rightarrow 0$ star	yes	Chain linearity. Linear perfluoroalkyl chains score ≈ 1; highly branched structures score lower.
per group	`mean_eccentricity`	N_G	float≥0	$\bar{\epsilon} = 1/\lvert V \rvert \sum_v \epsilon(v)$	yes	Average topological reach. Large for long linear chains; small for compact rings. Correlated with chain-length-driven persistence.
per group	`median_eccentricity`	N_G	float≥0	$\tilde{\epsilon} = \text{median}_v\, \epsilon(v)$	yes	Robust eccentricity estimate; less sensitive to a single terminal atom.

per group	`diameter`	N_G	int≥0	$d = \max_{u,v} d(u, v)$	yes	Longest shortest path. Tracks chain length directly for linear structures ($d = \|V\| - 1$).
per group	`radius`	N_G	int≥0	$r = \min_v \epsilon(v)$	yes	Minimum eccentricity. Low for compact symmetric fragments; complements the diameter for shape.
per group	`effective_graph_resistance`	N_G	float≥0	$\Omega = \sum_{i<j} r_{ij}$ (Kirchhoff index)	yes	Global chain topology. Large for long linear chains; smaller for branched/cyclic. Best single graph metric for group discrimination; used in preset `'best'`.
per group	`effective_graph_resistance_BDE`	N_G	float≥0	$\Omega_{\mathrm{BDE}} = \sum_{i<j} r_{ij}^{(\mathrm{BDE})}$, $w_{ij} = \mathrm{BDE}_{ij}/\mathrm{BDE}_{CC}$	yes	Bond-strength weighted Kirchhoff index. Encodes C–F bond weakness relative to C–C backbone; distinguishes highly fluorinated chains.
per group	`component_fraction`	N_G	float [0,1]	$f = \|V'\|/\|M\|$, V' = expanded component (incl. F/H)	yes	The fraction of the molecule covered by this fragment. Approaches 1 for highly fluorinated molecules (e.g., PFOA).
per group	`min_dist_to_center`	N_G	int≥0	$\min_{v\in S, c\in \mathrm{center}(G)} d(v, c)$	yes	Proximity of matched functional-group atoms to the graph center. Zero when the group is central; large for chain termini.
per group	`max_dist_to_periphery`	N_G	int≥0	$\max_{v\in S, p\in \mathrm{periphery}(G)} d(v, p)$	yes	Extension toward peripheral atoms. Encodes whether the group is internally embedded vs terminally exposed.
per group	`min_dist_to_barycenter`	N_G	int≥0	$\min_{v\in S, b\in \mathrm{bary}(G)} d(v, b)$	yes	Distance to the topological center of mass. Complements the center distance for asymmetric or multi-functional molecules.
molecule	`n_components`	1	int≥0	$N_C = \|\{(g, i)\}\|$	—	Total structural complexity. Distinguishes mono-functional from oligomeric/multi-head PFAS.
molecule	`total_size`	1	int≥0	$S = \sum_{g,i} \|C_{g,i}\|$	—	Cumulative halogenated carbon count. Correlated with the MW of the halogenated fraction.
molecule	`mean_size`	1	float≥0	$\bar{s} = S/N_C$	—	Average fragment size. Distinguishes many short vs. few long chains at equal total halogenation.

molecule	max_size	1	int≥0	$s_{\max} = \max_{g,i} \lvert C_{g,i} \rvert$	—	Size of the largest fragment. Primary driver of persistence and bioaccumulation.
molecule	mean_branching	1	float [0,1]	$\bar{\beta} = N_C^{-1} \sum_i \beta_i$	—	Average chain linearity across all components.
molecule	max_branching	1	float [0,1]	$\beta_{\max} = \max_i \beta_i$	—	Linearity of the most linear component; useful for mixed chain-type molecules.
molecule	mean_eccentricity	1	float≥0	$N_C^{-1} \sum_i \bar{\epsilon}_i$	—	Chain-length proxy. Independent of which groups were matched.
molecule	max_diameter	1	int≥0	$d_{\max} = \max_i d_i$	—	Diameter of the longest fragment. Directly tracks the longest halogenated chain.
molecule	mean_component_fraction	1	float [0,1]	$\bar{f} = N_C^{-1} \sum_i f_i$	—	Average fraction each fragment spans. Distinguishes localized vs. distributed halogenation.
molecule	max_component_fraction	1	float [0,1]	$f_{\max} = \max_i f_i$	—	Fraction covered by dominant fragment. Approaches 1 for heavily fluorinated molecules.

S4.2. Computational implementation

Algorithm architecture

The `ComponentsSolver` class orchestrates metric computation:

1. **Initialization** (`__init__`): Converts the input molecule into a *NetworkX* graph, identifies fluorinated components via `get_fluorinated_subgraph` (SMARTS matching followed by connected-component detection), and initializes the metric cache.
2. **Metric precomputation** (`_precompute_component_metrics`): Computes all graph metrics for each component and stores the results in `_component_metrics_cache` using `frozenset` keys.
3. **On-demand retrieval** (`compute_component_metrics`): Returns cached metrics or computes them if not available. Handles edge cases such as single-node components and disconnected graphs.

Complexity analysis

The computational complexity of per-component metric evaluation is as follows:

- **Eccentricity computation**: $\mathcal{O}(n \cdot (n + m))$ using `nx.eccentricity`, which performs BFS from each node. For molecular graphs where $m \approx 4n$, this simplifies to $\mathcal{O}(n^2)$.
- **Barycenter computation**: $\mathcal{O}(n \cdot (n + m)) \approx \mathcal{O}(n^2)$, based on all-pairs shortest paths.
- **Effective resistance computation**: $\mathcal{O}(n^2 \cdot (n + m)) \approx \mathcal{O}(n^3)$ in the worst case, but reduced in practice to $\mathcal{O}(n^2)$ via early termination for large components.
- **SMARTS distance metrics**: $\mathcal{O}(k \cdot p \cdot (n + m))$, where $k = |S_{match}|$ and $p = |B(G_C)| + |Z(G_C)| + |P(G_C)|$. Since $p \propto n$, this scales as $\mathcal{O}(k \cdot n^2)$.

The dominant contribution arises from SMARTS distance computations repeated across PFAS groups ($G \approx 117$), resulting in an overall scaling of $\mathcal{O}(n^2)$, consistent with benchmark results (Section S8).

Caching strategy

Metrics are computed once per component and cached using `frozenset` representations as immutable keys. This avoids redundant computation when a single component matches multiple groups. Cache invalidation occurs upon object destruction (`__exit__`).

Performance optimization

Two parameters control computational cost:

- `compute_component_metrics`: If set to `False`, disables all graph-metric calculations and returns `NaN` values.
- `limit_effective_graph_resistance`: Controls computation of effective resistance. Setting this parameter to `0` disables resistance calculations.

S4.3. `PFASEmbedding` and `PFASEmbeddingSet`

The `PFASEmbedding` module converts the structured output of the classification pipeline (Sections S2–S3) into fixed-width numerical vectors suitable for machine learning (ML), clustering, and dimensionality reduction. The resulting representation integrates group-level classification with component-level structural descriptors (Section S4.1), enabling both categorical and topological characterization of PFAS.

Two public classes are provided:
- **`PFASEmbedding`:** representation of a single molecule or dataset as a *NumPy*[7] `ndarray` subclass
- **`PFASEmbeddingSet`:** collection of embeddings with associated metadata for batch operations

Representation design

The embedding is constructed as a concatenation of feature blocks derived from two complementary sources:
1. **Group-level encoding**: captures the presence, multiplicity, and size of structural motifs defined in the group classification scheme (Section S3).
2. **Component-level graph metrics**: capture the topology and spatial organization of halogenated components identified via SMARTS matching and graph decomposition (Sections S2 and S4.1).

This two-layer representation links chemical functionality (group definitions) with molecular topology (graph metrics), allowing structurally distinct PFAS with similar functional group compositions to be distinguished.

`PFASEmbedding` object

`PFASEmbedding` is a subclass of `numpy.ndarray`, returned by `to_array()` on a `PFASEmbeddingSet`. As it inherits from `ndarray`, all standard *NumPy* operations (indexing, broadcasting, arithmetic) apply directly.

In addition to the underlying array, the object includes:
- **`group_names`**: list of feature names, including metric suffixes where applicable
- **`smiles`**: list of SMILES strings indexing rows
- **Dimensionality reduction methods**: `perform_pca()`, `perform_tsne()`, `perform_umap()`
- **Distribution comparison**: `compare_kld()` for KL-divergence between datasets

Multiple encoding blocks can be combined by passing a list of mode names to the `component_metrics` parameter; the simplest case is a single-element list such as `['binary']`.

Encoding modes

The `component_metrics` parameter controls how group-level information is encoded. Each selected mode contributes a block of n_{groups} features. These encodings operate on components derived from SMARTS-based matching and graph decomposition (Section S2), and therefore reflect the structural units identified during classification.

The available encoding modes represent increasing levels of structural detail:

- **`binary`** (default): indicates whether at least one component matching the group is present. This provides a minimal representation of group membership and is suitable for coarse classification, but does not distinguish multiplicity, size, or topology.
- **`count`**: encodes the number of matched components per group, capturing functional multiplicity and distinguishing mono-functional from multi-functional or oligomeric structures.

Additional size-based encodings:

- **`max_component`**: size of the largest component associated with each group, serving as a proxy for dominant chain length and correlating with persistence and bioaccumulation.
- **`total_component`**: sum of component sizes per group, capturing the total halogenated carbon burden for each functional class.

Graph-based descriptors (see Table S3 and Section S4) provide continuous, structure-sensitive features:

- **`effective_graph_resistance`**: Kirchhoff index of the component, capturing global connectivity and path redundancy
- **`branching_index`**: degree of branching, with values approaching 1 for linear chains
- **`min_dist_to_centre`**: minimum distance between matched functional-group atoms and the component center

For these descriptors, the aggregation method across multiple components per group can be specified (default: `mean`).

In contrast to `binary` and `count` encodings, graph-based metrics enable discrimination between molecules with similar functional group composition but different topologies.

Molecule-level descriptors

The `molecule_metrics` parameter appends global descriptors computed across all components (Section S4.1), including:

- `total_size`
- `mean_size`
- `max_size`
- `mean_branching`

- `max_branching`
- `max_diameter`
- `mean_component_fraction`

These features capture overall molecular complexity and complement the group-resolved representation.

Group selection and presets

The `group_selection` parameter determines which groups (Section S3) are included:

- '**all**' (default): all 117 groups (all groups except aggregate group, ID 119, and the targeted halogen group, e.g., fluorine group, ID 48, when `halogens='F'`)
- '**oecd**': 28 OECD-type groups (IDs 1–28).
- '**generic**': 48 core generic functional groups (IDs 29–76).
- '**telomers**': 42 fluorotelomer groups (IDs 77–118), excluding the aggregate group (ID 119).
- '**generic+telomers**': 89 combined groups

Predefined configurations are available via the `preset` parameter (e.g., '`best`', '`binary`', '`count`'), each specifying optimized combinations of `component_metrics` and `molecule_metrics`.

Multi-halogen stacking

When multiple halogens are specified (Section S1), e.g., `halogens=['F','Cl']`, the fingerprint/embedding is constructed by performing independent parsing for each halogen and concatenating the resulting feature blocks.

S5. PFAS regulatory definitions

In addition to functional group classification (Section S3), `PFASGroups` also evaluates a compound's compliance with major regulatory PFAS definitions. Table S4 summarizes the implemented definitions and their structural criteria in simplified form.

SMARTS patterns for the various regulatory initiatives were derived from published graphical representations and textual descriptions. For complex definitions, particularly the EU Restriction Proposal[8], custom SMARTS patterns were developed and validated against dedicated accuracy and specificity test sets. Each definition was validated using curated positive and negative test sets provided in the GitHub repository (PFASGroups/data/PFAS_definitions_smarts.json).

Table S4. PFAS regulatory definitions

Definition name	Structural criteria (summary)
OECD definition	≥1 CF_2 or CF_3 unit without H, Cl, Br, or I
EU PFAS restriction	OECD-based definition with additional exclusion rules on substituents
UK PFAS definition	≥1 CF_3 (no H/Cl/Br/I) or ≥2 contiguous CF_2 units
OPPT	≥2 fluorinated carbons without hydrogen; extended structural motifs (3 patterns)
PFASSTRUCTv5	Defined structural motifs (4 patterns) or fluorine ratio threshold

S5.1. OECD definition

The Organisation for Economic Co-operation and Development (OECD) defines PFAS as fluorinated substances containing at least one fully fluorinated methyl (–CF_3) or methylene (–CF_2–) carbon atom without any hydrogen, chlorine, bromine, or iodine substituents.[9]
The corresponding SMARTS pattern (see Figure S1) is:
`[#6X4!$({[#6H1]})!$({[#6][#17,#35,#53]})(F)F`

This pattern matches tetra-coordinated carbon atoms that: (1) have no bonded H atoms, (2) are not bonded to Cl, Br, or I, and (3) are bonded to at least two F atoms.

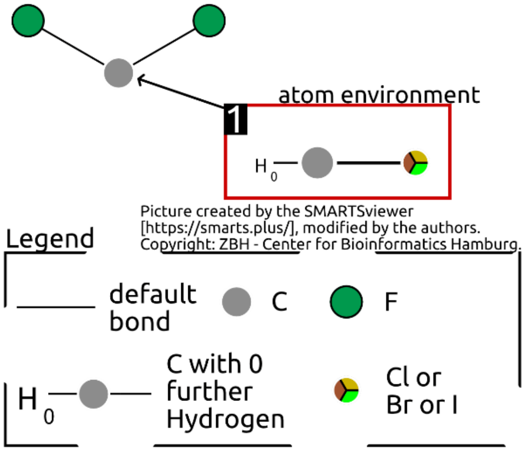

Figure S1. Representation of the SMARTS pattern used to match the OECD definition[9]. The figure is created using the `SMARTSviewer` (`smarts.plus`[10]) from ZBH - Center for Bioinformatics Hamburg.

S5.2. EU Restriction Proposal

The EU Restriction Proposal[8] builds on the OECD definition, requiring the presence of either CF_3–X or X–CF_2–X′, where no bonds to hydrogen, chlorine, bromine, or iodine are allowed. Additional exclusion criteria apply:

A substance is excluded if it matches only CF_3–X or X–CF_2–X′ where:
- X is either –OR or –NRR′, and
- X′ is either methyl (–CH_3), methylene (–CH_2–), an aromatic group, a carbonyl group, –OR″, –SR″ or –NR″R‴

with R, R′, R″, and R‴ being hydrogen, methyl, methylene, aromatic group, or carbonyl groups.

To implement this definition, two SMARTS patterns are used:
- CF_3–X pattern:

`[#6X4!$([#6][#8H1,#8$([#8][#6H3,#6H2X4,a,#6X3$([#6]=[#8])])])!$([#6][#7H2,#7H1$([#7][#6H3,#6H2X4,a,#6X3$([#6]=[#8])]),#7$([#7]([#6H3,#6H2X4,a,#6X3$([#6]=[#8])])[#6H3,#6H2X4,a,#6X3$([#6]=[#8])])])!$([#6H1])!$([#6][#17,#35,#53])](F)(F)F`

- X–CF_2–X′ pattern:

`[#6X4!$([#6]([#8H1,#8$([#8][#6H3,#6H2X4,a,#6X3$([#6]=[#8])])])[#6H3,#6H2X4,#6X3$([#6]=[#8]),a,#8H1,#8$([#8][#6H3,#6H2X4,a,#6X3$([#6]=[#8])]),#16$([#16H1,#16$([#16][#6H3,#6H2X4,a,#6X3$([#6]=[#8])])]),#7$([#7H2,#7H1$([#7$([#7][#6H3]),#7$([#7][#6H2X4]),#7a,#6X3$([#6]=[#8])]),#7$([#7]([#6H3,#6H2X4,a,#6X3$([#6]=[#8])])[#6H3,#6H2X4,a,#6X3$([#6]=[#8])])])])!$([#6]([#7H2,#7H1$([#7][#6H3,#6H2X4,a,#6X3$([#6]=[#8])]),#7$([#7]([#6H3,#6H2X4,a,#6X3$([#6]=[#8])])[#6H3,#6H2X4,a,#6X3$([#6]=[#8])])])[#6H3,#6H2X4,#6X3$([#6]=[#8]),a,#8H1,#8$([#8][#6H3,#6H2X4,a,#6X3$([#6]=[#8])]),#16$([#16H1,#16$([#16][#6H3,#6H2X4,a,#6X3$([#6]=[#8])])]),#7$([#7H2,#7H1$([#7][#6H3,#6H2X4,a,#6X3$([#6]=[#8])]),#7$([#7]([#6H3,#6H2X4,a,#6X3$([#6]=[#8])])[#6H3,#6H2X4,a,#6X3$([#6]=[#8])])])])!$([#6H1])!$([#6][#17,#35,#53])](F)F`

S5.3. UK Regulatory Management Option Analysis (RMOA)

The UK RMOA (2023)[11,12] defines PFAS as substances containing either:
- at least one fully fluorinated methyl group (CF_3) without hydrogen or other halogens:
  `F[#6H0X4!$([#6][#17,#35,#53])](F)F`, or
- at least two contiguous perfluorinated methylene groups:
  (`F[#6](F)[#6X4](F)(F)`).

S5.4. EPA's Office of Pollution Prevention and Toxics (OPPT) definition

The OPPT definition requires at least two carbon atoms that are fluorinated and contain no hydrogen substituents[13,14]. The updated 2023 definition[13] introduces additional structural motifs, represented by the following SMARTS patterns:

`[#6H0X4](F)(F)[#6H0X4](F)`

`[#6](F)(F)([F,#8,#6H0X4])[#8][#6](F)(F)[F,#8,#6H0X4]`

`[#6](F)(F)(F)[#6]([#6](F)(F)F)([F,#6H0X4])[F,#6H0X4]`

Figure S2 provides graphical representations of these patterns.

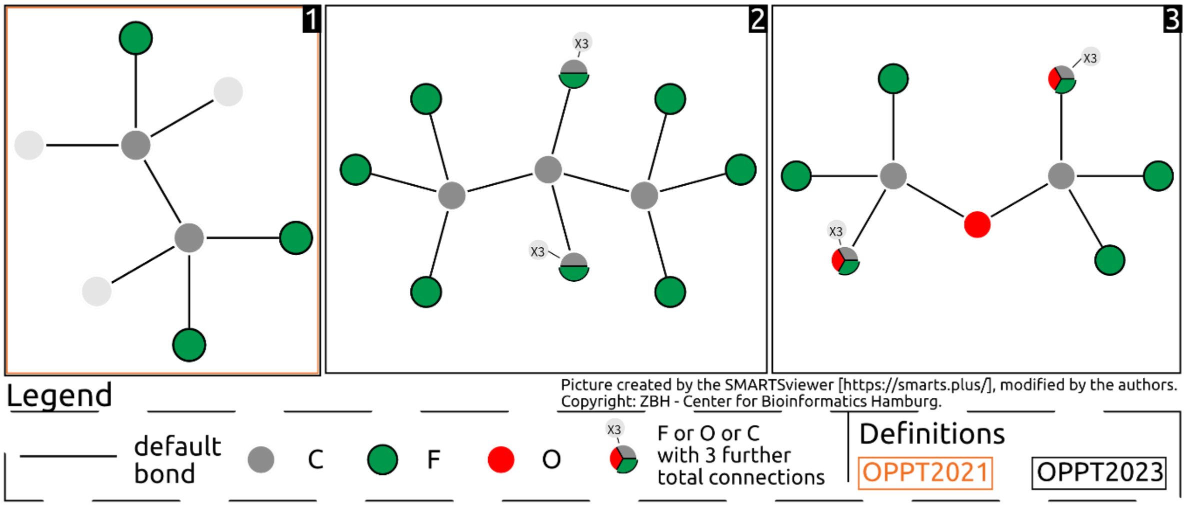

Figure S2. Representation of the SMARTS pattern used to match the OPPT definitions[13]. The figure is created using the `SMARTSviewer` (`smarts.plus`[10]) from ZBH - Center for Bioinformatics Hamburg.

S5.5. PFASSTRUCTv5

PFASSTRUCTv5 was developed by Gaines et al.[15] and combines structural pattern matching with a fluorine ratio criterion. A compound satisfies the definition if it matches any of the four structural patterns or has a fluorine ratio greater than 0.3. The structural criteria are presented graphically in Gaines et al. and were transcribed here into SMARTS patterns. The resulting patterns are listed below, with their graphical representation shown in Figure S3.

SMARTS patterns:

```
F[#6](F)(F)[#6](F)
F[#6](F)[#6](F)(F)
F[#6](F)(F)[#6]~{}[#6](F)(F)
[#6](F)(F)[#5,#7,#8,#14,#15,#16][#6](F)F
```

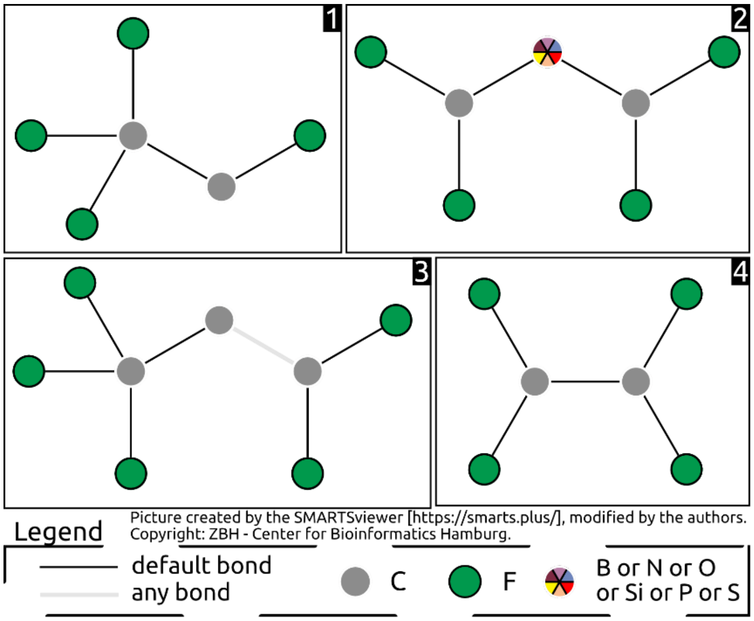

Figure S3. Representation of the SMARTS pattern used to match PFASSTRUCTv5[15]. The figure is created using the `SMARTSviewer` (`smarts.plus`[10]) from ZBH - Center for Bioinformatics Hamburg.

The accuracy of the implemented SMARTS patterns was evaluated against the PFASSTRUCTv5 (https://comptox.epa.gov/dashboard/chemical-lists/PFASSTRUCTV5) list from DSSTox (downloaded as an SDF v3000 file, version August 2022). According to Gaines et al., the fluorine ratio is computed based on molecular mass excluding hydrogen atoms. Of the 14,725 compounds in the dataset, all but seven (<0.05%) were correctly classified.

- Three compounds could not be parsed by *RDKit* from the SDF v3000 file and could not be recovered via PubChem or CASRN (five additional problematic entries were successfully retrieved via PubChem/CASRN and correctly classified).
- Two compounds contain wildcard atoms (DTXSID60183181 and DTXSID60957127) and were therefore not assessed.
- Two compounds are inconsistent with the PFASSTRUCTv5 definition, as they match neither the structural patterns nor the fluorine ratio criterion:
  - DTXSID70745890: `Nc1c[13cH]13c[13cH]c1`, fluorine ratio = 0.25 (excluding hydrogen and ignoring carbon-13)
  - DTXSID20745840: `N[13c]113c[13cH][13cH][13cH][13cH]1F`, fluorine ratio = 0.22 (excluding hydrogen and ignoring carbon-13)

S6. Molecule prioritization

`PFASGroups` provides a dedicated prioritization module (`prioritise.py`) for ranking molecules based on either their structural similarity to a reference set or their intrinsic fluorinated-component characteristics. Prioritization is performed using the `prioritise_molecules` function.

Two complementary strategies are implemented.

S6.1. Reference-based prioritization

When a reference set is provided, each candidate molecule is scored based on its similarity to the reference set in the embedding space (Section S4.3).

Specifically, each molecule is represented by its fingerprint vector (embedding) f_i, and the reference set is represented by the mean fingerprint vector (embedding):

$$s_i = \frac{\mathrm{f}_i \cdot \bar{\mathrm{f}}_{\mathrm{ref}}}{\parallel \mathrm{f}_i \parallel \parallel \bar{\mathrm{f}}_{\mathrm{ref}} \parallel},$$

where f_i is the embedding vector of the candidate molecule i and $\bar{\mathrm{f}}_{\mathrm{ref}}$ is the mean embedding over all reference molecules. Scores lie in $[0,1]$; a score of 1 indicates an identical group-activation profile to the reference centroid. Candidates with no group matches receive a score of 0. The embedding encoding used for similarity calculation is controlled by the `group_selection` and `count_mode` parameters (Section S4.3). By default, all groups are included (`'all'`) and the `max_component` encoding is used, providing sensitivity to dominant chain length.

S6.2. Intrinsic prioritization

When no reference is provided, molecules are ranked by a weighted combination of their total fluorinated component ratio and the size of their largest components.

S7. Software environment

PFASGroups is compatible with *Python* 3.8+ and *RDKit* 2020.03+; however, all results reported in this study were generated using the specific software versions listed in Table S5.

The module was developed and tested on both Microsoft Windows 11 and Ubuntu 24.04 systems. Fingerprints used for the comparison between PFASGroups and TxP_PFAS were generated on *System 1* (Table S5). Bayesian hierarchical models for fingerprint comparison were executed on *System 2a*. The PFAS-Atlas benchmark required a separate conda environment (*System 2b*) due to integer overflow incompatibilities between the MHFP module and *NumPy* 2.x.

Table S5. Hardware and software characteristics and versions.

	**System 1**	**System 2a**	**System 2b**
**Operating System**	Windows 11 Home 25H2<br>OS build 26200.8037	Ubuntu 24.04.4 LTS<br>Kernel Linux 6.17.0-19-generic	Ubuntu 24.04.4 LTS<br>Kernel Linux 6.17.0-19-generic
**OS type**	64 bits ARM	64 bits	64 bits
**Processor**	Qualcomm Snapdragon X Elite 3.42 GHz	Intel Core i7-7700HQ 2.8 GHz	Intel Core i7-7700HQ 2.8 GHz
**Integrated Graphics**	Qualcomm Adreno X1-85	Intel Graphics 630	Intel Graphics 630
**Graphics Processing Unit**	–	NVIDIA GeForce GTX 1050 Ti	NVIDIA GeForce GTX 1050 Ti
**Memory**	32	16Go	16Go
**Hardware model**	ASUSTek A14 UX	ASUSTek UX550VE	ASUSTek UX550VE
**Conda distribution**	Miniforge3	Miniforge3	Miniforge3
**Mamba version**	2.3.3	2.1.1	2.1.1
**Environment name**	chem	stan	pfasatlas
**Python**	3.14.2	3.9.23	3.9.23
**RDKit**	2025.09.3	–	2025.09.2
**NumPy**	2.3.5	1.26.4	1.26.4
**NetworkX**	3.6.1	–	3.2.1
**pandas**	3.0.0	2.3.1	2.3.1
**scikit-learn**	1.8.0	–	1.6.1
**SciPy**	1.16.3	1.12.0	1.13.1
**matplotlib**	3.10.8	3.9.4	3.9.4
**pystan**	–	2.19.1.1	–

S8. Benchmarking and validation

This section evaluates PFASGroups through (i) computational complexity analysis, (ii) large-scale classification comparison against PFAS-Atlas using halogenated compounds from the ECHA Classification and Labelling Inventory (C&L inventory; $n = 28{,}328$)[16], (iii) structural validation on PubChem fluorotelomers[17], and (iv) predictive benchmarking on ToxCast toxicological endpoints[18].

S8.1. Computational complexity

Timing benchmarks (2,500 molecules, 5 iterations, 9–621 non-hydrogen atoms, chain lengths from 5–200 carbon atoms) confirm quadratic scaling, $\mathcal{O}(n^2)$. A quadratic model was selected over linear, exponential, and log-linear alternatives based on AIC, BIC, and R^2 (Figure S4, Table S6).

With all metrics enabled, execution time follows: $t = 0.006114\, n^2 + 18.98$ ms.

Disabling all component metrics reduces the quadratic coefficient by approximately 20%: $t = 0.004876\, n^2$ (Figure S5).

On the C&L inventory benchmark ($n = 28{,}328$; Intel i7-7700HQ), PFASGroups averages 23.2 ms per molecule (±19.98 ms, median 17.5 ms, 95th percentile 54.4 ms).

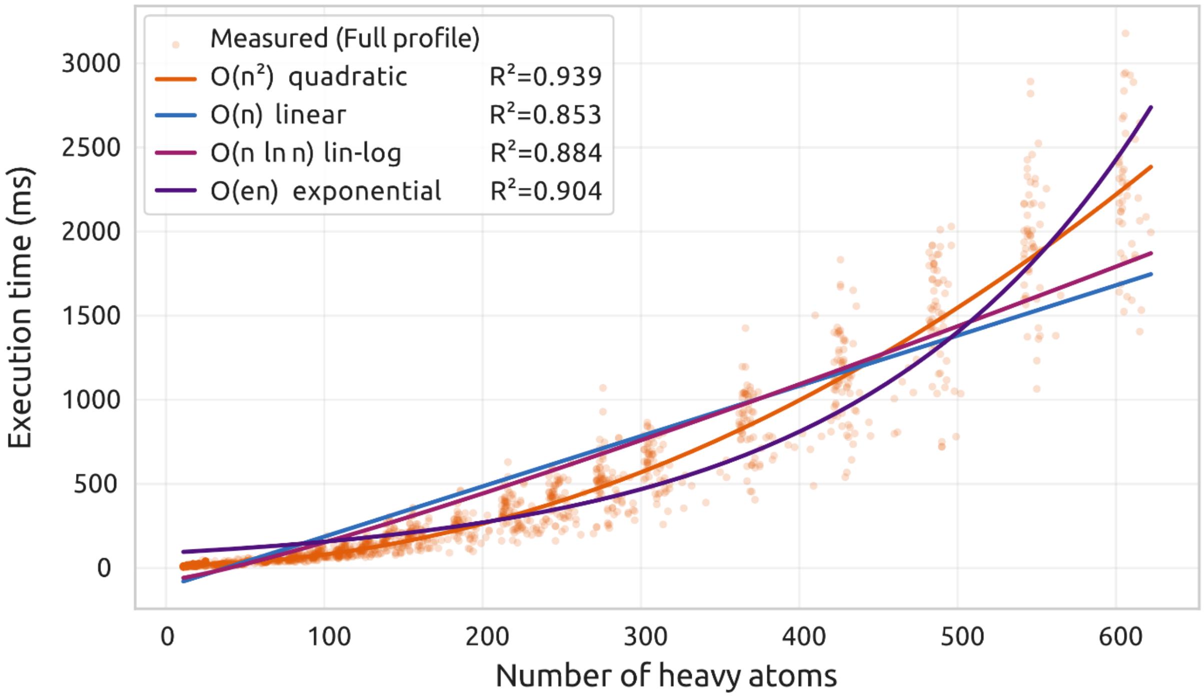

Figure S4. Comparison of computational complexity models for PFASGroups using 2,500 stress-benchmark molecules with all component metrics enabled ('Full' profile). The coefficient of determination (R^2) is reported for each model. The quadratic model provides the best overall fit ($R^2 \approx 0.939$) while remaining interpretable, outperforming linear, log-linear, and exponential alternatives.

Table S6. Comparison of computational complexity models across three timing profiles. The quadratic model (bold) provides the best fit in all cases (R^2 = 0.91–0.94) and yields the lowest AIC and BIC values. '`Full`' includes all graph metrics (including effective graph resistance); '`No EGR`' excludes effective graph resistance; '`No metrics`' excludes all component-level graph metrics. Parameters *a* and *b* denote the fitted coefficients of each model.

profile	model	complexity	equation	R^2	AIC	BIC	*a*	*b*
**Full**	**Quadratic**	$\mathcal{O}(n^2)$	$a \cdot n^2 + b$	**0.939**	**31288**	**31299**	**$6.114 \cdot 10^{-3}$**	**$1.898e \cdot 10^{1}$**
	Linear	$\mathcal{O}(n)$	$a \cdot n + b$	0.853	33480	33492	2.988	$-1.121 \cdot 10^{2}$
	Log-linear	$\mathcal{O}(n \log(n))$	$a \cdot n \log(n) + b$	0.884	32896	32908	$4.853 \cdot 10^{1}$	$-7.118 \cdot 10^{1}$
	Exponential	$\mathcal{O}(\exp^n)$	$a \cdot \exp^{b \cdot n}$	0.904	32414	32426	$9.044 \cdot 10^{1}$	$5.483 \cdot 10^{-3}$
**No EGR**	**Quadratic**	$\mathcal{O}(n^2)$	$a \cdot n^2 + b$	**0.924**	**32037**	**32049**	**$6.109 \cdot 10^{-3}$**	**$1.502 \cdot 10^{1}$**
	Linear	$\mathcal{O}(n)$	$a \cdot n + b$	0.832	34024	34036	3.035	$-1.174 \cdot 10^{2}$
	Log-linear	$\mathcal{O}(n \log(n))$	$a \cdot n \log(n) + b$	0.863	33512	33524	$4.917 \cdot 10^{-1}$	$-7.567 \cdot 10^{1}$
	Exponential	$\mathcal{O}(\exp^n)$	$a \cdot exp^{b \cdot n}$	0.898	32783	32795	$8.568 \cdot 10^{1}$	$5.556 \cdot 10^{-3}$
**No metrics**	**Quadratic**	$\mathcal{O}(n^2)$	$a \cdot n^2 + b$	**0.911**	**31318**	**31330**	**$4.876 \cdot 10^{-3}$**	**1.824**
	Linear	$\mathcal{O}(n)$	$a \cdot n + b$	0.788	33492	33503	2.379	$-9.953 \cdot 10^{1}$
	Log-linear	$\mathcal{O}(n \log(n))$	$a \cdot n \log(n) + b$	0.824	33022	33034	$3.870 \cdot 10^{-1}$	$-6.774 \cdot 10^{1}$
	Exponential	$\mathcal{O}(\exp^n)$	$a \cdot exp^{b \cdot n}$	0.900	31618	31630	$5.656 \cdot 10^{1}$	$5.904 \cdot 10^{-3}$

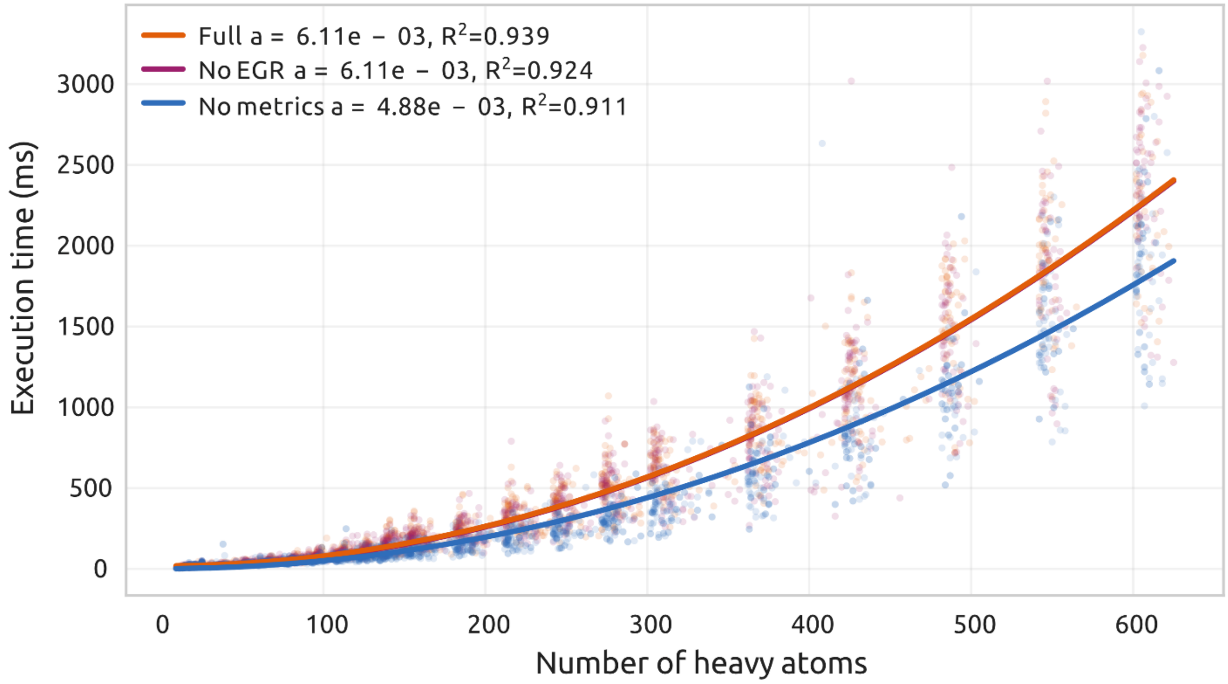

Figure S5. Effect of graph metrics on `PFASGroups` execution time across 2,500 molecules (9–619 non-H atoms). Quadratic fits are shown for three profiles: '`Full`' (all metrics, $a = 6.11 \times 10^{-3}\,\mathrm{ms} \cdot \mathrm{atom}^{-2}$), '`No EGR`' (effective graph resistance excluded; $a = 6.11 \times 10^{-3}$), and '`No metrics`' (all component metrics disabled; $a = 4.88 \times 10^{-3}$). Disabling all component metrics reduces the quadratic scaling coefficient by approximately 20%.

S8.2. Comparison with PFAS-Atlas

`PFASGroups` was compared with `PFAS-Atlas`[19,20] on:

- the OECD 2018 PFAS dataset[2]
- 28,328 halogenated molecules from the C&L inventory[16]
- a randomly generated stress dataset of large molecules

Speed Comparison

`PFASGroups` is slightly faster on average for the OECD dataset but slower for the C&L inventory and the stress dataset (Figure S6). This difference arises from:

- larger molecule sizes
- the computational cost of graph-based metrics

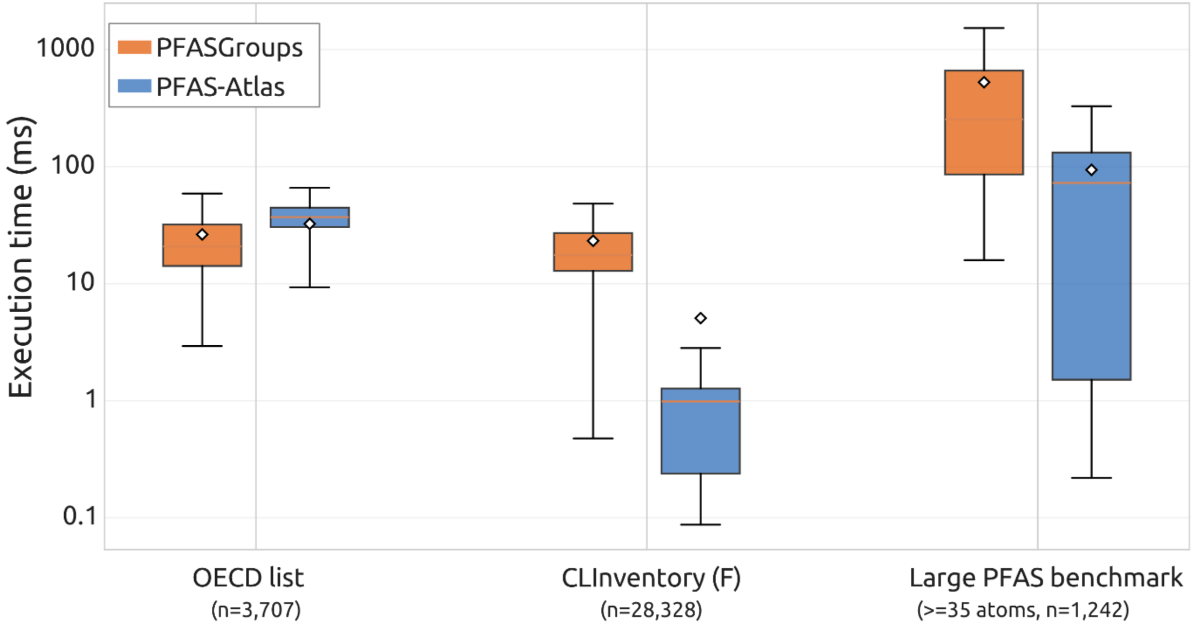

Figure S6. Execution time distributions for `PFASGroups` (orange) and `PFAS-Atlas` (blue) across three datasets: the OECD 2018 list ($n = 3{,}707$), the fluorinated subset of the C&L Inventory ($n = 28{,}328$), and a large-molecule stress benchmark (≥35 heavy atoms, $n = 1{,}242$). Boxes represent the interquartile range, and diamonds indicate the mean. A logarithmic scale is used due to the wide range of execution times across datasets.

Classification agreement

Agreement between `PFASGroups` and `PFAS-Atlas` on the OECD 2018 dataset is high (Figure S7), with most discrepancies arising from differences in classification strategy. `PFASGroups` allows multiple group assignments per compound, whereas `PFAS-Atlas` assigns each compound to a single class, which inherently limits one-to-one correspondence between the two systems.

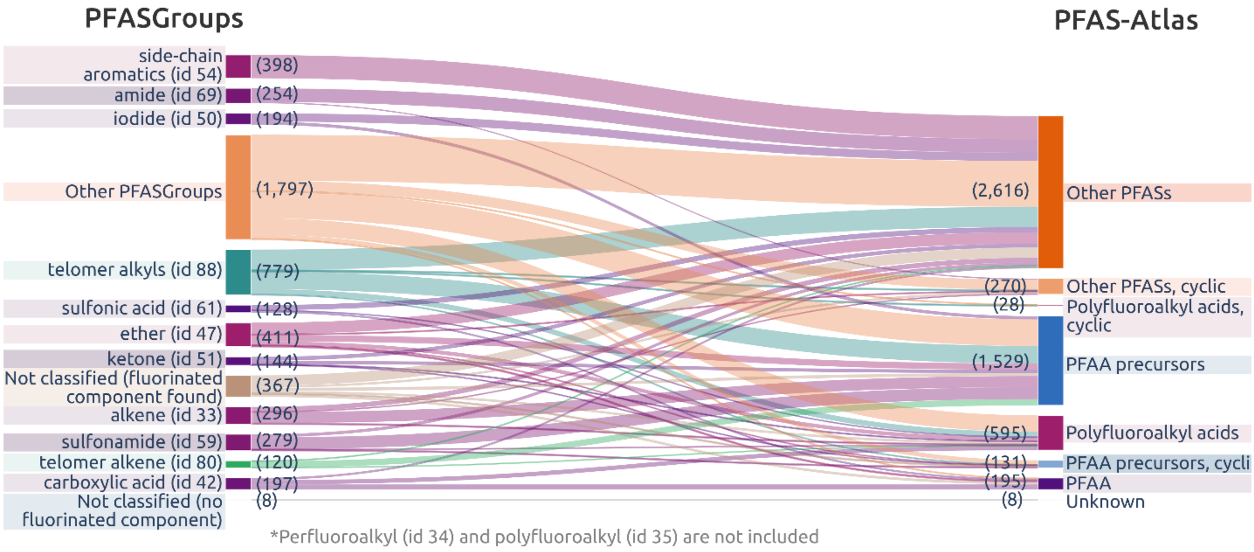

Figure S7. Sankey diagram comparing PFASGroups generic and telomer groups assignments (IDs 29-118, excluding IDs 34 and 35) with `PFAS-Atlas` class-1 assignments for the OECD 2018 dataset. The 12 most frequent PFASGroups categories are shown individually, while all remaining groups are aggregated under "`Other PFASGroups`".

On the C&L Inventory dataset, overall agreement reaches 77.7% (Table S7). The majority of disagreements stem from systematic differences in structural scope. PFASGroups identifies 6,216 compounds as PFAS that are not detected by `PFAS-Atlas`. All of these are fluorinated compounds, including 3,856 containing only fluorine and 2,360 containing additional chlorine or bromine atoms. These cases primarily arise from the broader polyhalogenated group definitions implemented in PFASGroups, which allow detection of minimally fluorinated structures such as mono-fluorinated aromatics, cyclic systems, and short alkyl chains that do not meet the stricter polyfluoroalkyl criteria used by `PFAS-Atlas`.

Table S7. Classification agreement between PFASGroups and `PFAS-Atlas` for fluorine-containing compounds in the C&L Inventory ($n = 28{,}328$).

	**`PFAS-Atlas`: PFAS**	**`PFAS-Atlas`: Non-PFAS**
**PFASGroups: PFAS**	20,735 (73.2%)	6,216 (21.9%)
**PFASGroups: Non-PFAS**	113 (0.4%)	1,264 (4.5%)

Conversely, `PFAS-Atlas` uniquely identifies 113 compounds that are not detected by PFASGroups. These compounds are predominantly vinylic or gem-difluoroalkene structures, in which fluorine atoms are bound to sp^2-hybridised carbons. Such structures fall outside the PFASGroups definition of polyfluoroalkyl components, which is limited to sp^3-carbon chains. Representative examples of these disagreements are shown in Figure S8.

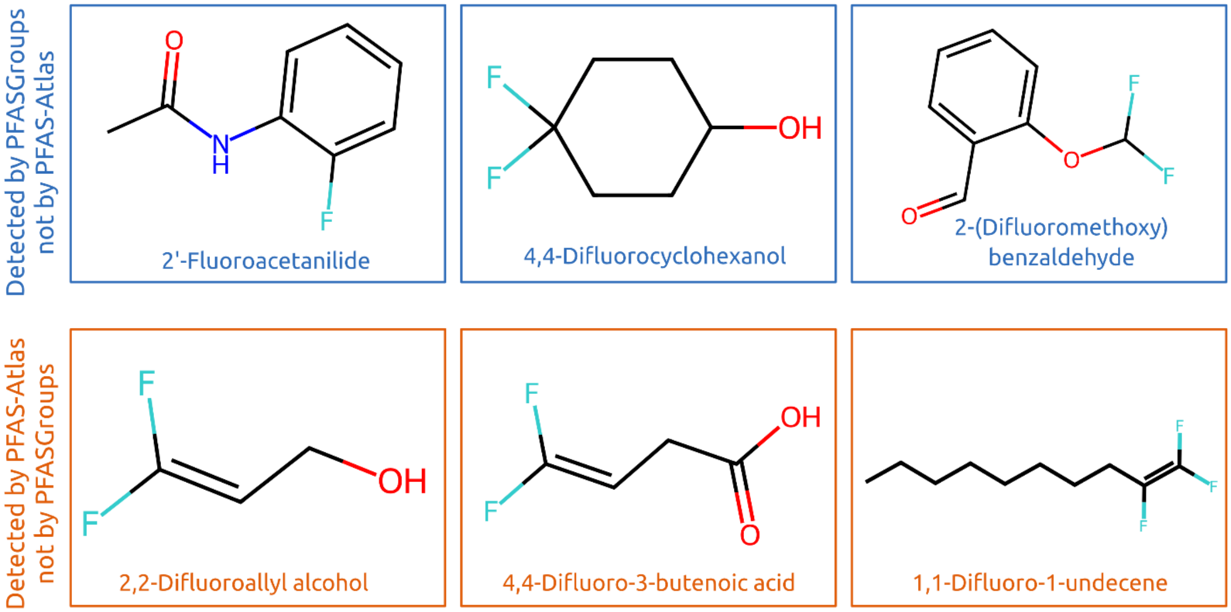

Figure S8. Representative examples of classification disagreements between `PFASGroups` and `PFAS-Atlas`. The top row shows compounds detected by `PFASGroups` as containing per- or polyfluorinated components but not classified as PFAS by `PFAS-Atlas`. The bottom row shows compounds classified as PFAS by `PFAS-Atlas` but not retained by `PFASGroups`.

Highly branched structures

To evaluate the handling of highly branched fluorinated compounds, a dataset of 300 structures was generated using a custom Python script (function `generate_random_mol` in `generate_mol.py`). Carbon backbones were constructed iteratively by adding carbon atoms while respecting valence constraints for an initial set of atoms, followed by the random addition of further carbons until a target branching index was reached. The resulting backbones were then per- or polyfluorinated by substituting all or a proportion of hydrogen atoms with fluorine. A functional group was subsequently attached at a randomly selected position by replacing a hydrogen or fluorine atom, and any remaining hydrogen at the attachment site was substituted with fluorine.

For this benchmark, only carboxylic acids (group 42) and sulfonic acids (group 61) were introduced, allowing direct comparison with expected `PFAS-Atlas` classifications. A successful classification by `PFASGroups` was defined as the correct identification of the exact functional group ID, whereas `PFAS-Atlas` was considered successful if any class other than "`Other PFASs`" or "`Unknown`" was assigned. The distribution of branching indices for the generated dataset is shown in Figure S9.

`PFASGroups` correctly identified the functional group in all cases, whereas `PFAS-Atlas` failed to assign the expected group in 11 of 300 molecules, primarily due to complex branching patterns that obscured the functional group environment. In addition, a subset of molecules failed at least one PFAS definition, most commonly the OPPT

definition, reflecting the sensitivity of certain regulatory criteria to highly branched topologies. Representative examples illustrating these cases are provided in Figure S9.

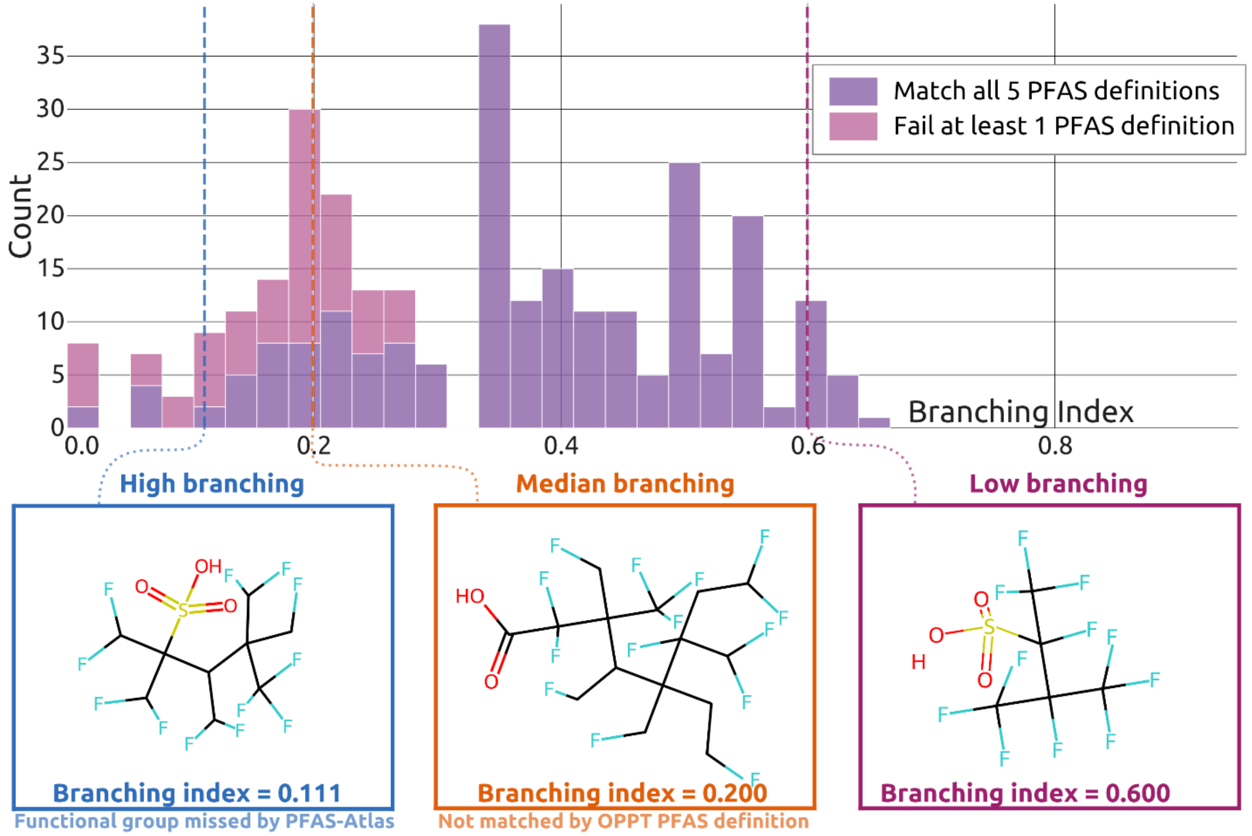

Figure S9. Evaluation on a synthetic dataset of highly branched fluorinated compounds ($n = 300$). The distribution of the branching index is shown, stratified by compliance with the implemented PFAS definitions (all definitions satisfied vs at least one definition failed, most often the OPPT definition). Representative structures illustrate key edge cases: a highly branched molecule for which `PFAS-Atlas` fails to assign the expected functional group, and a molecule excluded by at least one PFAS definition due to its branching topology.

Specificity assessment

Specificity was evaluated using the OECD 2018 dataset by analyzing co-detections of functional groups and identifying cases of unexpected overlap. To distinguish chemically meaningful co-detections from false positives, a hierarchical dependency network was defined comprising 41 nodes and 44 directed edges, encoding expected relationships between groups. For example, detection of an OECD perfluoroalkyl carboxylic acid (ID 6) necessarily implies detection of the corresponding generic carboxylic acid (ID 42).

Observed group co-occurrences were compared against this network to identify violations of the expected hierarchy. After accounting for all permitted dependencies, no unexpected group co-detections were observed. This corresponds to a specificity of 100%, indicating that the SMARTS patterns and associated constraints do not trigger

spurious functional group assignments beyond those justified by structural relationships.

S8.3. PubChem fluorotelomer validation

To evaluate the detection of fluorotelomer-specific groups (IDs 77–118), `PFASGroups` was applied to a dataset of 785 fluorotelomer compounds retrieved from PubChem using the keyword "fluorotelomer". The dataset covers a broad range of structures, including fluorotelomer alcohols, carboxylic acids, sulfonamides, phosphate esters, betaines, and other commercially relevant derivatives.

`PFASGroups` correctly identified 734 of 785 molecules as fluorotelomers, yielding a detection rate of 93.5%. The 51 undetected compounds predominantly comprise short-chain or structurally atypical molecules that do not meet the CH_2 linker constraint required for fluorotelomer classification. Among the detected molecules, an average of 2.5 fluorotelomer groups was assigned per compound, reflecting the frequent occurrence of multiple functional motifs within commercial fluorotelomer structures.

The distribution of detected groups and the most frequently (top 20) assigned fluorotelomer classes are summarized in Figure S10.

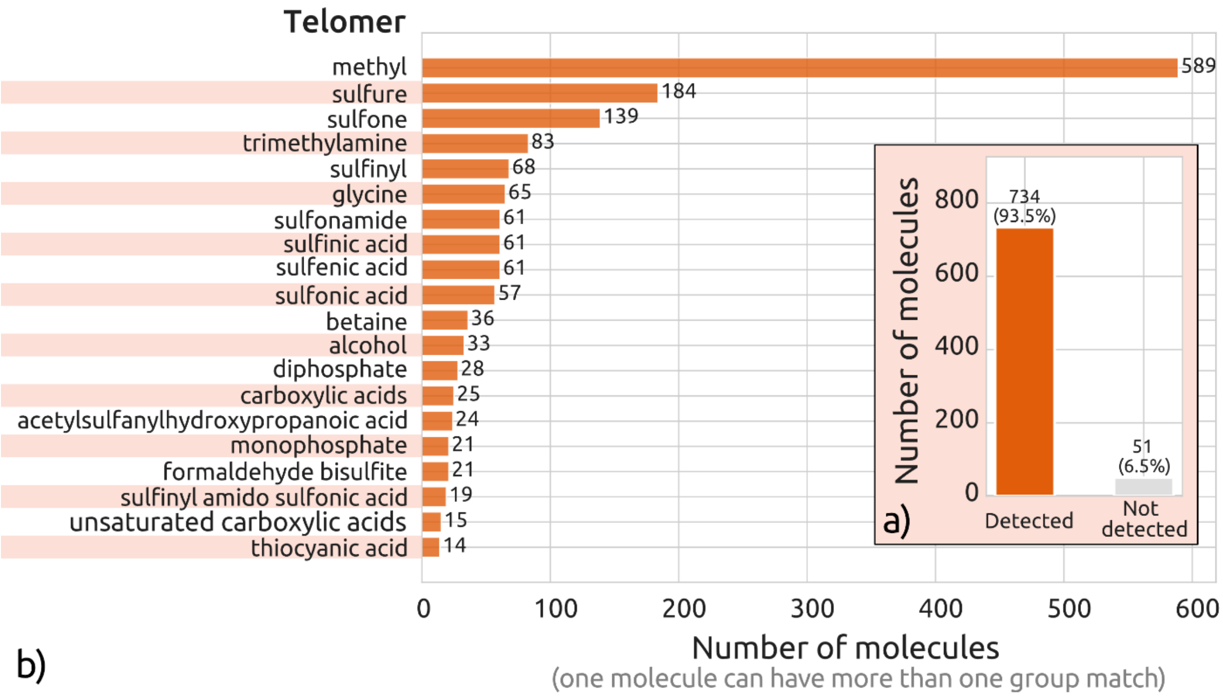

Figure S10. Validation of fluorotelomer detection against PubChem data ($n = 785$). (**a**) Detected and undetected molecules, corresponding to a 93.5% detection rate. Undetected cases ($n = 51$) predominantly comprise short-chain or structurally atypical compounds that do not satisfy the CH_2 linker constraint. (**b**) Top 20 most frequently detected fluorotelomer groups, with "telomer methyl" (589 molecules) being the most prevalent, consistent with the widespread occurrence of the C_nF_{2n+1}–CH_2– backbone in fluorotelomers.

S8.4. Validation of PFAS definitions

The five implemented PFAS definitions (OECD, EU PFAS Restriction Proposal, OPPT 2023, UK RMOA, and PFASSTRUCTv5) were validated using curated positive and negative test sets embedded in the JSON configuration file (`PFAS_definitions_smarts.json`). These test sets comprise representative structures that are expected to be included (positives) or excluded (negatives) according to each respective definition.

For each definition, SMARTS-based queries were applied to the test sets, and the resulting classifications were compared against the expected labels. This procedure enabled systematic verification of both sensitivity (correct identification of positives) and specificity (correct exclusion of negatives), ensuring that the implemented SMARTS patterns accurately reproduce the intended structural criteria.

For PFASSTRUCTv5, additional validation was performed using an external reference dataset (see Section S5.5), including evaluation of both substructure-based matching and the fluorine ratio criterion. Together, these tests confirm that all five definitions are implemented consistently and behave as expected across a diverse set of PFAS and non-PFAS structures.

S8.5. Fingerprint benchmarking on ToxCast endpoints

Data source and experimental protocol

Toxicological endpoint data were obtained from the U.S. EPA ToxCast invitroDB v4.3 database[18,21]. Chemical structures were retrieved using DTXSID identifiers via the PubChem REST API[17].

Fifteen endpoints spanning nuclear receptor activity, cytochrome P450 inhibition, cytotoxicity, and genotoxicity were selected. Data from level 5 (table *mc5*) were used, corresponding to curve-fitted AC_{50} values, which were subsequently binarized using the hit-call flag (active/inactive)[22,23].

A subset of approximately 808 organofluorine compounds was extracted to enable direct comparison between PFAS-specific structural encodings. These included the 129-bit `ToxPrint` PFAS fingerprint (`TxP_PFAS`)[24] and multiple `PFASGroups` embedding configurations capturing group presence, component size, graph-based metrics such as effective graph resistance, and molecule-wide descriptors.

Classification models were trained using repeated stratified *k*-fold cross-validation ($k = 3$, 5 repeats; 15 folds per endpoint). Two algorithms were evaluated: Histogram Gradient Boosting and Random Forest. Hyperparameters were optimized exclusively on the training partitions using an inner 3-fold grid search to prevent information leakage. Performance is reported as mean ± standard deviation across all cross-validation folds. Unless stated otherwise, results correspond to the Gradient Boosting model, which consistently performed slightly better than Random Forest.

All computations were performed on *System 1* described in Table S5. The `TxP_PFAS` and `ToxPrint` structural fingerprint vectors were pre-generated using ToxPrint Chemotypes v2.0-rev711 (Molecular Networks GmbH 2014) (https://github.com/mn-am/toxprint/releases). A local copy of ToxCast invitrodb v4.3 downloaded from EPA Clowder (Feshuk 2024) was served via MariaDB 12.1.2 and accessed via SQLAlchemy 2.0.44 with the mariadb connector 1.1.14, using `pandas` to extract endpoint data and corresponding binary activity labels.

Performance metrics and the effect of class imbalance

Four complementary metrics are reported for each endpoint and feature set[25]:

- **ROC-AUC**: area under the receiver operating characteristic curve; measures overall ranking ability independent of the classification threshold.
- **Average Precision (AP)**: area under the precision-recall curve; directly reflects performance on the minority (positive) class and is sensitive to class imbalance.
- **Balanced Accuracy**: arithmetic mean of sensitivity and specificity; corrects for class imbalance at the decision boundary.
- **Matthews Correlation Coefficient (MCC)**: a single summary statistic derived from all four entries of the confusion matrix and ranges from 0 for random predictions to 1 for perfect classification.

Across the evaluated endpoints, MCC values are consistently lower than ROC–AUC or balanced accuracy. This behavior is expected in imbalanced datasets, as the MCC denominator depends on the product of class marginals, leading to reduced absolute values when the proportion of active compounds is low (typically around 10% for several endpoints considered here). As a result, even models with good ranking performance can exhibit modest MCC values.

Despite this scaling effect, MCC remains one of the most informative metrics in imbalanced settings because it accounts for all confusion matrix components without favoring the majority class[25]. Its interpretation should therefore be considered in the context of the underlying class distribution rather than in absolute terms alone.

Overall performance

Table S8 summarizes the predictive performance of the Gradient Boosting model, averaged across all 15 endpoints (mean ± standard deviation). The best-performing `PFASGroups` configurations consistently outperform the `TxP_PFAS` fingerprint across all four evaluation metrics.

Table S8. Predictive performance on 15 ToxCast endpoints for organofluorine compounds ($n \approx 808$) using Gradient Boosting. Values are reported as mean ± standard deviation across endpoints. The best-performing configuration for each metric is highlighted in bold. 'mol' denotes molecule-wide graph metrics, 'branch' the branching index, and 'EGR' the effective graph resistance.

Algorithm	Feature Set Abbr.	ROC-AUC	Avg. Prec.	MCC	Bal. Acc.
TxP_PFAS	TxP_PFAS	0.618 ± 0.070	0.374 ± 0.179	0.174 ± 0.128	0.593 ± 0.063
PFASGroups	PFG_binary	0.600 ± 0.065	0.350 ± 0.177	0.148 ± 0.107	0.583 ± 0.063
PFASGroups	PFG_EGR	0.555 ± 0.056	0.303 ± 0.160	0.074 ± 0.083	0.541 ± 0.051
PFASGroups	PFG_EGR+mol	0.698 ± 0.079	0.457 ± 0.207	0.263 ± 0.115	0.651 ± 0.066
PFASGroups	PFG_EGR+branch+mol	0.698 ± 0.078	0.457 ± 0.206	0.263 ± 0.114	0.651 ± 0.066
PFASGroups	PFG_total_component+mol	**0.699 ± 0.077**	**0.463 ± 0.207**	**0.266 ± 0.113**	**0.652 ± 0.063**

The inclusion of molecule-wide graph metrics is the primary driver of performance gains. Augmenting structural encodings with these descriptors increases ROC–AUC by approximately 0.10 on average (e.g., from 0.600 for the binary fingerprint to 0.697 for binary+mol). In contrast, the choice of component-level encoding (binary versus EGR) has only a marginal effect when evaluated within the same feature set, with differences in ROC–AUC below 0.001.

The top-performing configuration, combining total component size with molecule-wide metrics (PFG_total_component+mol), achieves an average ROC–AUC of 0.699, compared to 0.618 for TxP_PFAS, resulting in an absolute improvement of 0.081. Similar trends are observed for average precision, balanced accuracy, and MCC, indicating that the performance gain is consistent across threshold-independent, threshold-dependent, and correlation-based metrics.

Overall, these results demonstrate that incorporating global structural descriptors substantially enhances the predictive power of PFAS-specific representations, while fine-grained variations in component-level encoding contribute comparatively little once molecule-level information is included.

Per-endpoint performance

Performance across individual endpoints is shown in Figure S11 for TxP_PFAS and the five best-performing PFASGroups configurations. Each endpoint is evaluated using all four metrics (ROC–AUC, average precision, MCC, and balanced accuracy), with the proportion of active compounds indicated in the subplot titles.

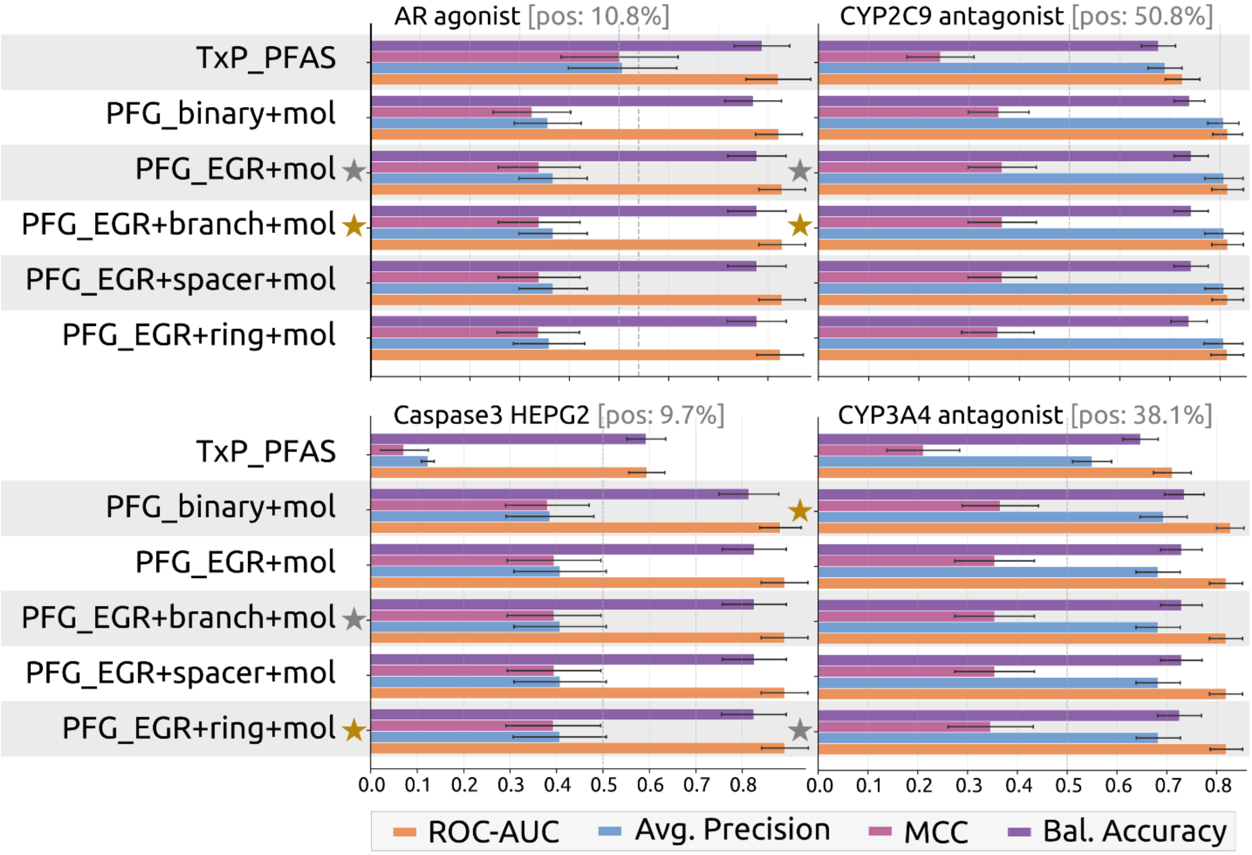

Figure S11. Per-endpoint predictive performance for TxP_PFAS and the five best-performing PFASGroups configurations on selected ToxCast invitroDB v4.3 endpoints. For each endpoint, four metrics are shown: ROC–AUC, average precision, Matthews correlation coefficient (MCC), and balanced accuracy. Values represent the mean across repeated cross-validation folds. The percentage of active compounds (positive class) is indicated in each subplot title. Gold and silver stars denote the best- and second-best-performing feature sets, respectively, based on ROC–AUC for each endpoint.

Across most endpoints, PFASGroups configurations that include molecule-wide metrics consistently outperform TxP_PFAS. The largest absolute improvement is observed for the Caspase-3 HEPG2 endpoint, where PFASGroups achieves substantially higher performance across all metrics. In contrast, for the AR agonist endpoint, although some PFASGroups configurations achieve slightly higher ROC–AUC values, the remaining metrics indicate more consistent performance for TxP_PFAS, highlighting endpoint-specific differences in model behavior.

To assess whether these differences are statistically meaningful while accounting for the dependence structure of repeated cross-validation, a Bayesian correlated *t*-test[26,27] was applied to fold-level performance differences between the best PFASGroups configuration and TxP_PFAS. The posterior distribution of the mean difference was estimated using a non-informative prior, with fold correlation accounted for via the Benavoli correction ($\rho = 1/n$). A Region of Practical Equivalence (ROPE) of ±0.01 was used to classify differences as negligible, and results are summarized as posterior

probabilities of PFASGroups outperforming, being equivalent to, or underperforming relative to TxP_PFAS (Figure S12).

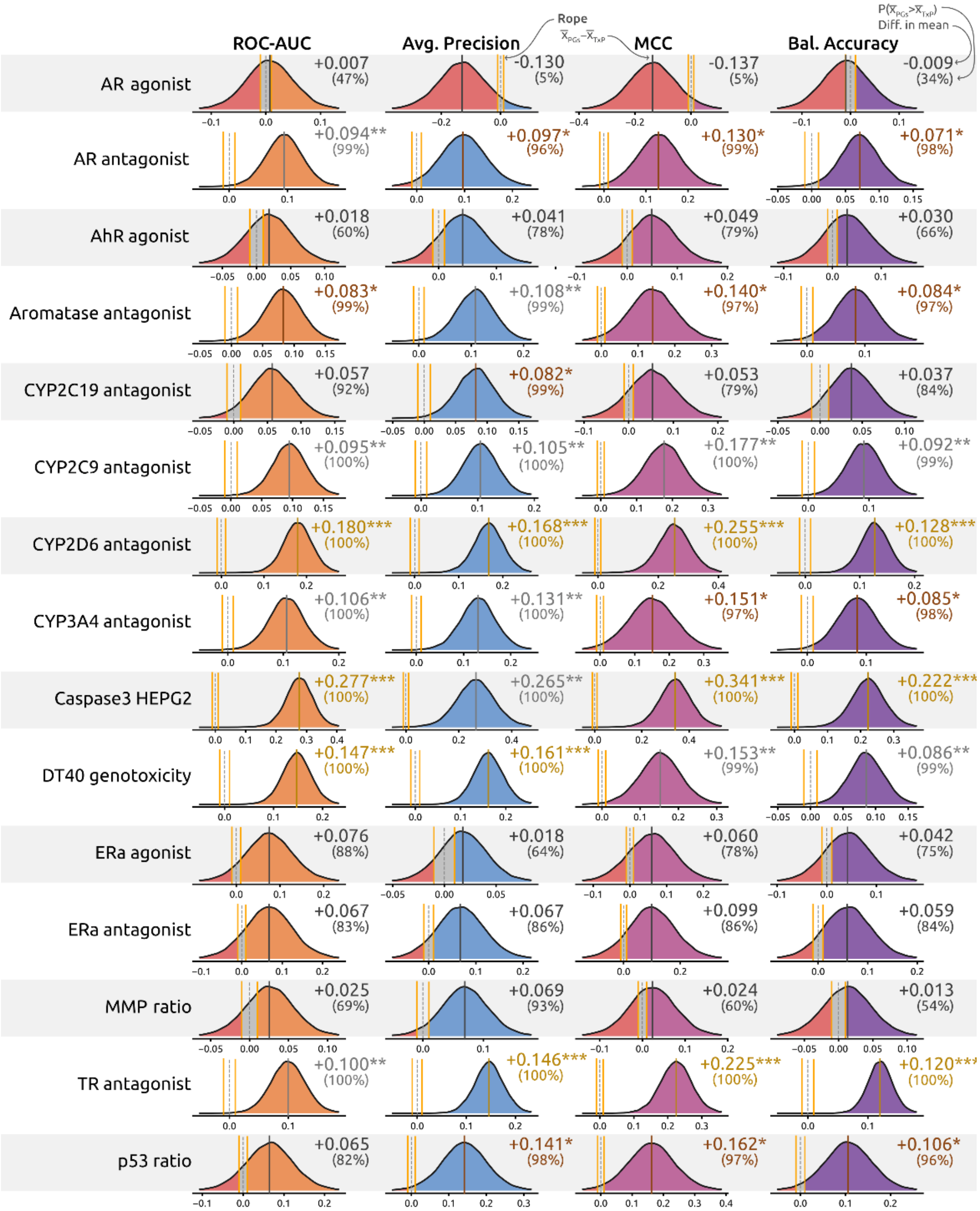

Figure S12. Posterior probability distributions of performance differences between the best PFASGroups configuration and TxP_PFAS for each ToxCast endpoint, estimated using the Bayesian correlated *t*-test[26]. Distributions are shown for four metrics: ROC–AUC (orange), average precision (blue), Matthews correlation coefficient (magenta), and balanced accuracy (violet). The region of practical equivalence (ROPE, ±0.01) is indicated by vertical lines. Central tendencies are marked, and statistical significance is annotated based on posterior probability (non-significant (black), * (95%, bronze), ** (99%, silver), *** (99.9%, gold)). Results account for the correlation between cross-validation folds following the Benavoli correction[26,27]. Red shading indicates superior performance of TxP_PFAS, grey indicates practical equivalence, and other colors indicate superior performance of models using PFASGroups embeddings. Plots were generated using PyStan following the methodology of Benavoli et al[26,27].

For 8 out of 15 endpoints, `PFASGroups` shows near-certain improvement in ROC–AUC (posterior probability ≥ 0.95). For the remaining endpoints, results are either inconclusive or indicate marginal differences between methods, with the AR agonist being the only case where `TxP_PFAS` is likely to perform better. Overall, these results confirm that the observed performance gains are robust across endpoints, while also highlighting endpoint-specific variability.

To further account for variation across endpoints, a hierarchical Bayesian model was applied following the framework of Benavoli et al[26,27]. This analysis, implemented using *PyStan* (*System 2a*, Table S5), yields consistent conclusions at the population level, supporting the overall superiority of `PFASGroups`-based representations (Figure S13).

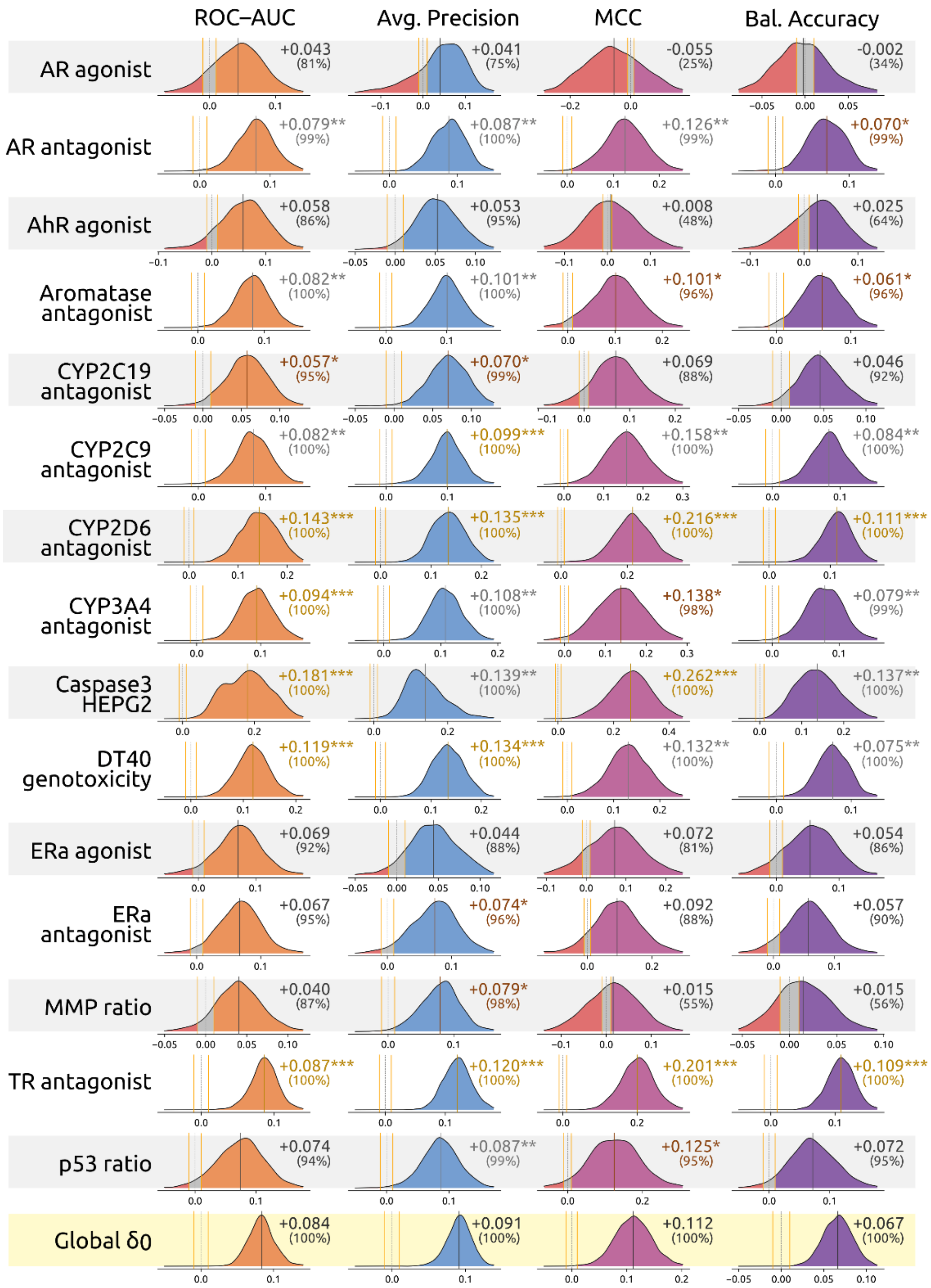

Figure S13. Posterior distributions from the hierarchical Bayesian model of performance differences between the best PFASGroups model and TxP_PFAS across ToxCast endpoints. Distributions are shown for ROC–AUC (orange), average precision (blue), Matthews correlation coefficient (magenta), and balanced accuracy (violet). The region of practical equivalence (ROPE, ±0.01) is indicated by vertical yellow lines, and central tendencies are shown as vertical lines. Effect sizes and posterior probabilities of PFASGroups outperforming TxP_PFAS are reported with significance levels indicated as non-significant (black), * (95%, bronze), ** (99%, silver), *** (99.9%, gold). Results account for the correlation between cross-validation folds following the Benavoli correction[26,27]. Red shading indicates superior performance of TxP_PFAS, grey indicates practical equivalence, and other colors indicate superior performance of models using PFASGroups embeddings. Computations were performed using PyStan (*System 2a*, Table S5) following the framework of Benavoli et al[26,27].

References

(1) Organisation for Economic Cooperation and Development (OECD), Environment Directorate. *Reconciling Terminology of the Universe of Per- and Polyfluoroalkyl Substances: Recommendations and Practical Guidance*; ENV/CBC/MONO(2021)25; Organisation for Economic Cooperation and Development: Paris, 2021. https://one.oecd.org/document/ENV/CBC/MONO(2021)25/En/pdf (accessed 2023-05-25).

(2) Wang, Z. S25 | OECDPFAS | List of PFAS from the OECD, 2018. https://doi.org/10.5281/zenodo.6349061.

(3) RDKit: Open-Source Cheminformatics. https://www.rdkit.org/ (accessed 2026-03-17).

(4) NetworkX. Network Analysis in Python. https://networkx.org/en/ (accessed 2026-03-17).

(5) Ellens, W.; Spieksma, F. M.; Van Mieghem, P.; Jamakovic, A.; Kooij, R. E. Effective Graph Resistance. *Spec. Issue Honor Dragos Cvetkovic* **2011**, *435* (10), 2491–2506. https://doi.org/10.1016/j.laa.2011.02.024.

(6) Hagberg, A. A.; Schult, D. A.; Swart, P. J. Exploring Network Structure, Dynamics, and Function Using NetworkX. In *Proceedings of the 7th Python in Science Conference*; Varoquaux, G., Vaught, T., Millman, J., Eds.; Pasadena, CA USA, 2008; pp 11–15.

(7) Harris, C. R.; Millman, K. J.; van der Walt, S. J.; Gommers, R.; Virtanen, P.; Cournapeau, D.; Wieser, E.; Taylor, J.; Berg, S.; Smith, N. J.; Kern, R.; Picus, M.; Hoyer, S.; van Kerkwijk, M. H.; Brett, M.; Haldane, A.; del Río, J. F.; Wiebe, M.; Peterson, P.; Gérard-Marchant, P.; Sheppard, K.; Reddy, T.; Weckesser, W.; Abbasi, H.; Gohlke, C.; Oliphant, T. E. Array Programming with NumPy. *Nature* **2020**, *585* (7825), 357–362. https://doi.org/10.1038/s41586-020-2649-2.

(8) European Chemicals Agency (ECHA). *Annex XV Restriction Report for the Restriction on the Manufacture, Placing on the Market and Use of PFASs.*; Version No. 2; 2023. https://echa.europa.eu/documents/10162/1c480180-ece9-1bdd-1eb8-0f3f8e7c0c49 (accessed 2023-10-11).

(9) Wang, Z.; Buser, A. M.; Cousins, I. T.; Demattio, S.; Drost, W.; Johansson, O.; Ohno, K.; Patlewicz, G.; Richard, A. M.; Walker, G. W.; White, G. S.; Leinala, E. A New OECD Definition for Per- and Polyfluoroalkyl Substances. *Environ. Sci. Technol.* **2021**. https://doi.org/10.1021/acs.est.1c06896.

(10) Ehrt, C.; Krause, B.; Schmidt, R.; Ehmki, E. S. R.; Rarey, M. SMARTS.plus – A Toolbox for Chemical Pattern Design. *Mol. Inform.* **2020**, *39* (12), 2000216. https://doi.org/10.1002/minf.202000216.

(11) UK's Health and Safety Executive (HSE). *UK REACH - PFAS RMOA - Call for evidence.* https://consultations.hse.gov.uk/crd-reach/pfas-rmoa-001/.

(12) UK's Health and Safety Executive (HSE); UK's Environment Agency. *Analysis of the Most Appropriate Regulatory Management Options (RMOA)*; 2023. https://www.hse.gov.uk/reach/assets/docs/pfas-rmoa.pdf.

(13) United States Environmental Protection Agency. Toxic Substances Control Act Reporting and Recordkeeping Requirements for Perfluoroalkyl and Polyfluoroalkyl Substances. *Fed. Regist.* **2023**, *88* (195), 70516–70559.

(14) Environmental Protection Agency. *Title 40–Protection of Environment*; 2023; Vol. 15 U.S.C. 2607(a)(7). https://www.ecfr.gov/current/title-40/part-705/section-705.3.
(15) Gaines, L. G. T.; Sinclair, G.; Williams, A. J. A Proposed Approach to Defining Per- and Polyfluoroalkyl Substances (PFAS) Based on Molecular Structure and Formula. *Integr. Environ. Assess. Manag.* **2023**, *19* (5), 1333–1347. https://doi.org/10.1002/ieam.4735.
(16) European Chemicals Agency (ECHA). Classification and Labelling (C&L) Inventory, 2024. https://echa.europa.eu/information-on-chemicals/cl-inventory-database.
(17) Kim, S.; Thiessen, P. A.; Bolton, E. E.; Chen, J.; Fu, G.; Gindulyte, A.; Han, L.; He, J.; He, S.; Shoemaker, B. A.; Wang, J.; Yu, B.; Zhang, J.; Bryant, S. H. PubChem Substance and Compound Databases. *Nucleic Acids Res.* **2016**, *44* (D1), D1202-1213. https://doi.org/10.1093/nar/gkv951.
(18) U.S. EPA. ToxCast & Tox21, 2026. https://www.epa.gov/chemical-research/toxicity-forecaster-toxcasttm-data.
(19) Su, A. PFAS-Atlas, 2024. https://github.com/su-group/PFAS-atlas.
(20) Su, A.; Cheng, Y.; Zhang, C.; Yang, Y.-F.; She, Y.-B.; Rajan, K. An Artificial Intelligence Platform for Automated PFAS Subgroup Classification: A Discovery Tool for PFAS Screening. *Sci. Total Environ.* **2024**, *921*, 171229. https://doi.org/10.1016/j.scitotenv.2024.171229.
(21) Feshuk, M. Invitrodb, 2024. https://clowder.edap-cluster.com/datasets/66b50344e4b0a7c65d2a0792?space=66858831e4b0a7c65d17841d.
(22) Filer, D. L.; Kothiya, P.; Setzer, R. W.; Judson, R. S.; Martin, M. T. Tcpl: The ToxCast Pipeline for High-Throughput Screening Data. *Bioinformatics* **2017**, *33* (4), 618–620. https://doi.org/10.1093/bioinformatics/btw680.
(23) National Center for Computational Toxicology, US EPA. *The ToxCast(TM) Analysis Pipeline(tcpl) An R Package for Processing and Modeling Chemical Screening Data (Version 2.0).* https://gaftp.epa.gov/COMPTOX/High_Throughput_Screening_Data/InVitroDB_V3.1/MySQL_Data/tcpl_v2_vignette.html.
(24) Richard, A. M.; Lougee, R.; Adams, M.; Hidle, H.; Yang, C.; Rathman, J.; Magdziarz, T.; Bienfait, B.; Williams, A. J.; Patlewicz, G. A New CSRML Structure-Based Fingerprint Method for Profiling and Categorizing Per- and Polyfluoroalkyl Substances (PFAS). *Chem. Res. Toxicol.* **2023**, *36* (3), 508–534. https://doi.org/10.1021/acs.chemrestox.2c00403.
(25) Chicco, D.; Tötsch, N.; Jurman, G. The Matthews Correlation Coefficient (MCC) Is More Reliable than Balanced Accuracy, Bookmaker Informedness, and Markedness in Two-Class Confusion Matrix Evaluation. *BioData Min.* **2021**, *14* (1), 13. https://doi.org/10.1186/s13040-021-00244-z.
(26) Benavoli, A.; Corani, G.; Demšar, J.; Zaffalon, M. Time for a Change: A Tutorial for Comparing Multiple Classifiers Through Bayesian Analysis. *J. Mach. Learn. Res.* **2017**, *18* (77), 1–36.
(27) Benavoli, A.; Corani, G.; Janez, D.; Dirks, M. BayesianTestsML. https://github.com/BayesianTestsML/tutorial/.
